# A Bottom‐Up Design Framework for Multifunctional Lattice Metamaterials

**DOI:** 10.1002/advs.202518923

**Published:** 2026-02-26

**Authors:** Zongxin Hu, Quanqing Tao, Junhao Ding, Shuo Qu, Haitao Ye, Jun Wei Chua, Tianxiao Niu, Rui Li, Winston Wai Shing Ma, Haoming Mo, Hui Liu, Wei Zhai, Xinwei Li, Xu Song

**Affiliations:** ^1^ Department of Mechanical and Automation Engineering Chinese University of Hong Kong Hong Kong SAR China; ^2^ Department of Mechanical Engineering National University of Singapore (NUS) Singapore Singapore; ^3^ Newcastle University in Singapore Faculty of Science, Agriculture, and Engineering Newcastle University Newcastle upon Tyne UK

**Keywords:** energy absorption, inverse design, lattice metamaterial, machine learning, multifunctional optimization

## Abstract

Driven by the demands of light weighting, multifunctionality has become increasingly important in the design of lattice metamaterials. While inverse design is crucial for developing such lattice structures, traditional inverse design methods such as topology optimization often fail to fully explore the design space. To overcome these limitations, this study introduces a generative AI framework that combines 3D Gaussian voxel generation with deep learning, enabling greater structural complexity and design freedom. As a proof of concept, we employ this bottom‐up design approach to shell lattice structures for optimized energy absorption and broadband sound absorption capabilities. A hybrid architecture combining a 3D convolutional neural network and a conditional deep convolutional generative adversarial network enables accurate energy absorption prediction and performance‐driven structural generation. In parallel, a genetic algorithm is employed to tune heterogeneous geometries for effective broadband sound absorption. Experimental validation through 3D‐printed stainless‐steel lattices demonstrates the superior multifunctionality of the designed structures —achieving 40%–200% greater energy absorption than conventional shell lattices, along with a high (average coefficient ∼0.7) and broad (α > 0.5 across 1000–5800 Hz) absorption bandwidth. Overall, our proposed framework overcomes the major drawbacks of existing inverse design approaches, offering enhanced voxel‐level model generation informed by physical insights.

## Graphical abstract

This study introduces a generative AI framework for designing multifunctional lattice metamaterials. The method combines 3D Gaussian voxel generation with deep learning, enabling greater design freedom and structural performance. The optimized lattice metamaterials achieve enhanced energy absorption by 40–200% compared to conventional structures and broadband sound absorption (α > 0.5, 1000‐–5800 Hz).

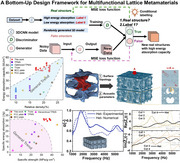

## 1 Introduction

Lattice metamaterials have garnered significant attention in engineering applications owing to their exceptional combination of high strength and lightweight characteristics [1, 2, 3]. These innovative materials exhibit remarkable mechanical properties that challenge conventional material paradigms, including but not limited to negative Poisson's ratio [4, 5], negative stiffness [6], negative elasticity [7, 8], and particularly impressive energy absorption capacity [1, 9]. Concurrently, the design philosophy of lattice structures has evolved toward multifunctional integration, demonstrating remarkable potential across diverse engineering disciplines [1]. These structures have shown extraordinary performance as advanced biomaterials for tissue engineering [10, 11, 12], precision optical devices with tunable properties [13, 14], and highly efficient thermal management systems with tailored heat transfer characteristics [15]. Among these multidisciplinary applications, the acoustic properties of lattice metamaterials have emerged as a particularly promising research frontier in recent years, driven by the growing demand for materials that can simultaneously deliver lightweight structural support and effective sound control [9, 16, 17]. In practical engineering contexts — such as aerospace, automotive, and building acoustics — there is a critical need for multifunctional components that not only meet mechanical performance requirements but also provide efficient acoustic properties within compact form factors.

Traditional design strategies for lattice metamaterials predominantly employ a forward design approach, which typically involves initial geometric modeling using implicit functions or explicitly using computer‐aided design (CAD) software, followed by extensive finite element method (FEM) simulations and experimental validation to assess their performances [18, 19, 20]. However, these conventional methods present two significant limitations: 1) heavy reliance on designers' empirical knowledge and prior experience, and 2) computationally expensive and time‐consuming iterative processes [21]. To address these challenges, increasing research attention has been directed toward inverse design methodologies in recent years. This paradigm shift enables performance‐driven topological optimization (TO) by establishing inverse mapping relationships between target mechanical properties and optimal geometric configurations, thereby offering greater design efficiency and precision [22]. Initial inverse design strategies for lattice structures are performed using various TO methods [23, 24]. Successes have been shown, for instance, to overcome the conventional Maxwell stability criterion [25], or to achieve optimized 3D geometric configurations [26]. Despite these, TO solutions exhibit considerable sensitivity to both boundary conditions and mesh parameters, which can substantially reduce the reliability of the results. Furthermore, a critical challenge in TO methods is their tendency to converge with local minima rather than the global solution [27, 28], leading to suboptimal structural designs.

In recent years, to overcome the limitations of traditional TO methods and enhance the design efficiency of complex structures, the application of machine learning (ML) techniques in lattice structure design has garnered increasing attention. Artificial neural networks (ANNs) have been used to predict the mechanical behavior of architected materials, for instance, to achieve forward prediction of stress–strain tensors in composite microstructures [29], to predict the stress–strain curves of lattice structures [22], and to study the mechanical response of heterogeneous lattices [30]. Beyond ANNs, convolutional neural networks (CNNs) also achieved breakthroughs in modeling the mechanical behavior of lattice structures [29, 30]. Despite this, these conventional deep learning methods have primarily been applied to performance prediction of structures, while the inverse generation of novel structures often requires assistance from other techniques such as genetic algorithms (GA) [21]. Efforts have been placed for the combined ANN and GA design of multifunctional lattice structures [9]. However, the approaches mentioned above suffer from two key limitations: a strong dependence on the quality of the training dataset and limited capacity to explore the boundaries of the design space. The limitations of these traditional ML approaches have spurred the adoption of more advanced generative models, particularly diffusion‐based models (DDPMs) and generative architectures, including Generative Adversarial Networks (GANs) [31] and Variational Autoencoders (VAEs) [32]. These models are better suited for generating novel structural configurations, a process commonly referred to as direct inverse design [21, 33]. DDPM have been utilized for the generation of novel high strength TPMS‐like lattice structures [33]. Additionally, VAEs have also been used to construct 2D optimal metamaterials for higher shear modulus and low Poisson's ratio [30]. However, diffusion models suffer from low sampling efficiency, requiring numerous iterative denoising steps (typically hundreds to thousands) to synthesize new structures. Moreover, VAEs, while more computationally efficient than diffusion models, exhibit inherent limitations in generative performance. Although GANs are known to potentially suffer from ‘mode collapse’ issues [33], GANs excel at producing realistic, high‐resolution samples, which is critical for designing functional structures or metamaterials. However, traditional GAN methods incorporate limited label information and are predominantly employed for generating models in 2D space. Consequently, further refinement is required to align these methods with the design objectives of our 3D lattice metamaterials.

Owing to the limitations of conventional GAN models, we herein introduce a conditional Deep Convolutional GAN (DCGAN) framework specifically tailored for the generation of multifunctional lattice metamaterials. By incorporating 3D deep convolutional layers with conditional inputs, the framework enables goal‐oriented generation of high‐resolution voxel‐based structures. Additionally, we integrate 3D Gaussian distribution‐based voxel modelling to overcome the geometric constraints of implicit function‐based methods, thereby significantly enhancing structural diversity and design freedom beyond traditional TPMS‐like architectures in shell lattices. Therefore, this work can be considered as the first truly Bottom‐Up Design Framework for Multifunctional Lattice Metamaterials.

## 2 Design Framework

### 2.1 Generation and Fabrication of 3D Gaussian Structures

The potential design space is composed of voxel blocks to fully expand the structural diversity to the greatest extent possible. On the other hand, the initial dataset for generative structures based on 3D Gaussian distributions is generated by the combination of implicit representations, guided by mathematical functions, which can be represented as [34]:

(1) $$f\left(x\right)=\frac{1}{2\pi^{\frac{3}{2}}{\left|\Sigma \right|}^{\frac{1}{2}}}\exp\left[-\frac{1}{2}{\left(x-\mu \right)}^{T}\Sigma^{-1}\left(x-\mu \right)\right]$$

where **x**  =  [*x*,  *y*,  *z*]^T^ and μ  = [μ_*x*_, μ_*y*_, μ_*z*_]^T^  denote the points and mean vectors (the central positions) of the Gaussian distributions, respectively. Then Σ is a 3  ×  3  ×  3 covariance matrix which describes the extension direction and amplitude of the distribution, which can be expressed as:

(2) $$\Sigma =\left[\begin{array}{ccc} \sigma_{x}^{2} & \sigma_{xy} & \sigma_{xz}\\ \sigma_{yx} & \sigma_{y}^{2} & \sigma_{yz}\\ \sigma_{zx} & \sigma_{zy} & \sigma_{z}^{2} \end{array}\right]$$

**FIGURE 1:**
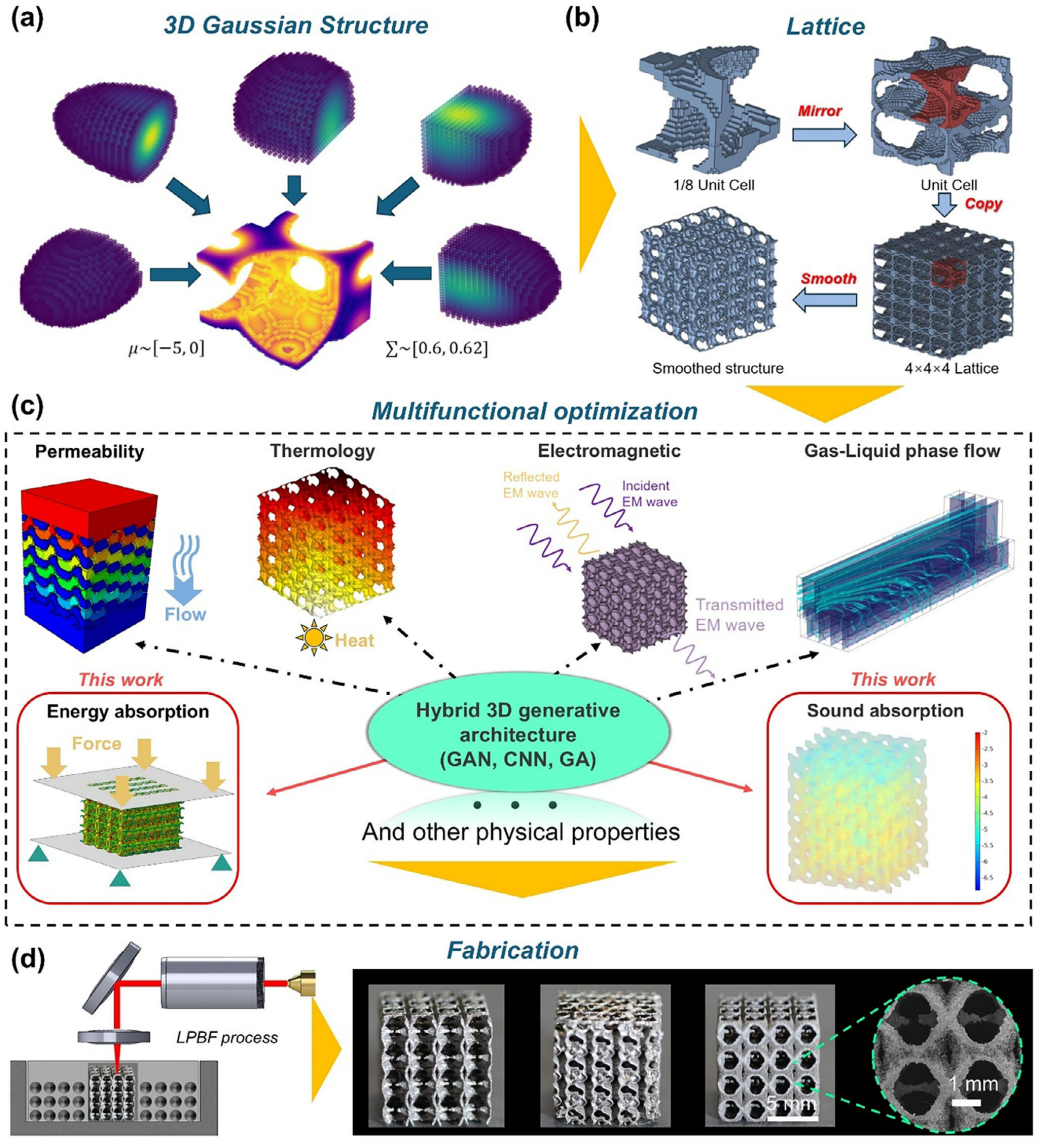
The overall design framework for multifunctional lattice metamaterials: (a) The process of generating a Gaussian surface lattice structure from five basic 3D Gaussian surfaces. (b)The process from generating a 1/8‐unit cell lattice structure to generating a complete manufacturable lattice structure and corresponding fabricated part by additive manufacturing. (c) The schematic of multifunctional tests and optimization process based on generated lattice structures. (d) The fabrication process for lattice structures with selected high energy and sound absorption capacities, and the SEM image for manufacturing quality.

The diagonal elements ($\sigma_{x}^{2},$ $\sigma_{y}^{2}$ and $\sigma_{z}^{2}$) represent the variances in three directions, which are also called the ‘fatness’ of the distribution. The other non‐diagonal elements represent the covariance between the variables, determining the rotation and correlation of the Gaussian distribution. As shown in Figure 1a, the 1/8‐unit cell of basic lattice structure is generated by the combination of five different Gaussian distributions. The design space of 1/8‐unit cell is set as [−5, 0] with 17 576 design voxels (each direction is divided into 26 voxels in the Euler coordinate systems). Besides, the ranges of values for mean vectors and covariance are [−5, 0] and [0.6, 0.62], respectively, which are determined by multiple attempts to maintain the continuity and boundary integrity. Meanwhile, to enhance the relevance and usability of the dataset, the relative density of the generated structures in this study was approximately maintained within the range of 17% to 23%, corresponding to a voxel block count between 2987 and 4043. A total of 20 000 diverse structures is generated as the training set for GAN models.

The generated 1/8‐unit cell is then transferred to the unit cell model through mirror operations in three directions as shown in Figure 1b. Meanwhile, in the field of metamaterial research [1], the performance analysis of a unit cell model is found to lack practical significance. Consequently, in this study, a 4 × 4 × 4 model, more suitable for engineering applications, is systematically derived through array operations based on the fundamental unit cell model. However, 3D models constructed using voxel blocks typically exhibit relatively coarse surfaces. Moreover, the quality of the resulting mesh is often suboptimal, which may compromise the manufacturability of the structure. To address this issue, we further refined the surface of the Gaussian model by applying the Laplace smoothing algorithm [35]:

(3) $${v_{i}}^{\prime }=v_{i}+\lambda \cdot \frac{1}{N}\sum_{j\in N(i)}\left(v_{j}-v_{i}\right)$$

where $v_{i}^{\prime }$ and v_*i*_ denote the vertices after and before the smoothing process, respectively. *N*(*i*) represent the adjacent vertices of the target vertex. Meanwhile, λ is the smoothing factor, and here we choose λ = 0.7 to maximize the preservation of the structural features. Meanwhile, these designed structures can be further integrated with the structural requirements of acoustic testing to obtain model shapes suitable for experimental verification, which will be elaborated in subsequent sections.

### 2.2 Multifunctional Optimization with Validation

The generated 3D Gaussian models are then optimized through considering different functional applications. Figure 1c presents several common performance‐oriented research topics in lattice structure investigations, where FEM‐based performance evaluation and optimization are systematically conducted. For instance, uniaxial compression tests employing FEM simulations are routinely performed to characterize the mechanical properties of metamaterials, with particular emphasis on their energy absorption capabilities. Furthermore, comprehensive assessments encompassing thermodynamic behavior, flow permeability, and electromagnetic performance can be achieved through carefully designed numerical simulations and experimental procedures, enabling cross‐verification of different lattice configurations. Among these functional characteristics, the inherent porosity of lattice structures renders them particularly suitable for sound absorption applications, as visually demonstrated in Figure 1c. Subsequently, both numerical simulation results and experimental data obtained can serve as a critical database for the hybrid bottom‐up design framework proposed in this study, providing substantial support for subsequent multi‐objective optimization processes. Various functional performance metrics can be strategically combined and investigated to ultimately achieve the desired multi‐functional optimization goals. To validate the reliability of the proposed multifunctional optimization framework, two representative cases – high energy absorption capacity and superior sound absorption efficiency – will be specifically selected as exemplary optimization targets for in‐depth investigation. Meanwhile, multifunctional lattice metamaterials based on 3D Gaussian designs are fabricated using high‐precision laser powder bed fusion (LPBF) (Figure 1d). For micro‐LPBF, powder particles may cause problems such as powder adhesion on the printing surface and insufficient melting [36]. In this work, the manufacturing process involves stainless steel 316L (SS316L) fine powder particles with diameters ranges from 5 to 25 µm with the D50 = 16.3 µm, and high resolution (25 µm) of laser beam to print lattices with the minimum feature size around 65.3 ±  4.6 µm. Our previous study has designed adaptive printing toolpaths that can effectively avoid the problem of insufficient melting and has been maturely applied to 316L stainless steel powder [37]. Moreover, scanning electron microscope (SEM) images in Figure S1 can demonstrate high surface quality for micro‐LPBF manufactured parts.

### 2.3 3D CNN Model for Predicting Energy Absorption

**FIGURE 2:**
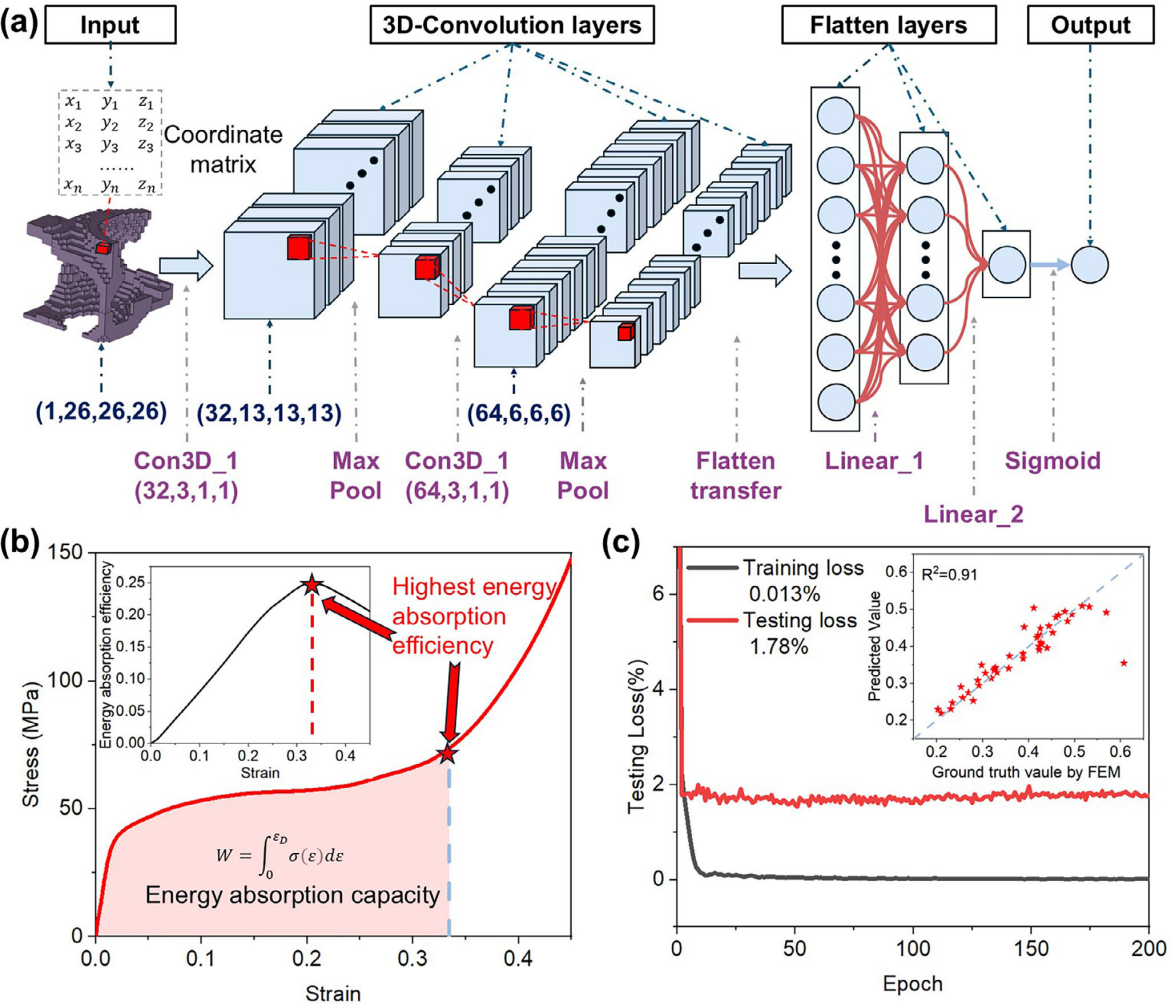
(a) The schematic of 3D CNN model. (b) The schematic of extracting the maximum energy absorption efficiency and energy absorption capacity from the stress–strain curve. (c) The training process and prediction accuracy of the CNN model.

The 3D CNN model [38], which requires a large amount of data points is performed to have an accurate prediction of energy absorption properties of generated lattice structures. As shown in Figure 2a, the structural information encapsulated within the 3D Gaussian model, specifically the coordinate matrix corresponding to each voxel block, will be extracted and utilized as the input information for the CNN model. The architecture of the input layer is (1, 26, 26, 26), which corresponds to the design degrees of freedom for 26 voxel blocks along each coordinate axis. Then architecture employs two sequential 3D convolutional layers for hierarchical feature extraction, each followed by 3D max‐pooling operations to reduce spatial dimensions while preserving critical structural patterns. The extracted features are flattened and processed through two fully connected (dense) layers for regression analysis. A sigmoid activation function finalizes the network output, normalizing predictions to a suitable range for energy absorption capacity. The 3D Gaussian models generated with smoothed 4  ×  4  ×  4 lattice structure are subjected to uniaxial compression tests to investigate their energy absorption performance. Then, based on the set up and results from experiments, the numerical models by FEM are used to establish the data set for the 3D CNN model. We then generate 1000 pairs of unique designs and corresponding stain‐stress curves. The energy absorption capacity *W*, which is the target output for CNN model as shown in Figure 2b, can be further obtained as:

(4) $$W=\int_{0}^{\varepsilon_{D}}\sigma \left(\varepsilon \right)d\varepsilon$$

where ε denotes the strain and σ(ε) represents the corresponding stress. Meanwhile, ε_*D*_ is the strain value when energy absorption efficiency reaches the maximum, which can be calculated as below [39]:

(5) $$\eta =\frac{1}{\sigma \left(\varepsilon \right)}\;\int_{0}^{\varepsilon }\sigma \left(\varepsilon \right)d\varepsilon$$

In addition, the dataset, which comprises 1000 individuals, will be partitioned into a training set and a test set in the ratio of 80% to 20%, respectively. Before the training process, the model's predicted target values, which represent the energy absorption capacity of each distinct structure, will be subjected to Min‐Max Normalization. This process linearly maps the data to the interval [0, 1], which can be calculated as:

(6) $$X_{norm}=\frac{X-X_{min}}{X_{max}-X_{min}}$$

where *X_min_* and *X_max_* denote the minimum and maximum values of the original data set, respectively, and *X_norm_* contains the new data points after normalization.

As shown in Figure 2c, trained with the mean squared error (MSE) loss function over 200 epochs, the 3D CNN model exhibited robust generalization performance and prediction accuracy, yielding a mere 2.008% error rate on the test dataset. Subsequently, the trained 3D CNN model was employed to predict the performance of other models in the dataset and perform label classification. Structures with an energy absorption capacity above 40 MPa were categorized as high energy‐absorption structures, while the others were labeled as low energy‐absorption structures.

### 2.4 3D Conditional DCGAN and GA

**FIGURE 3:**
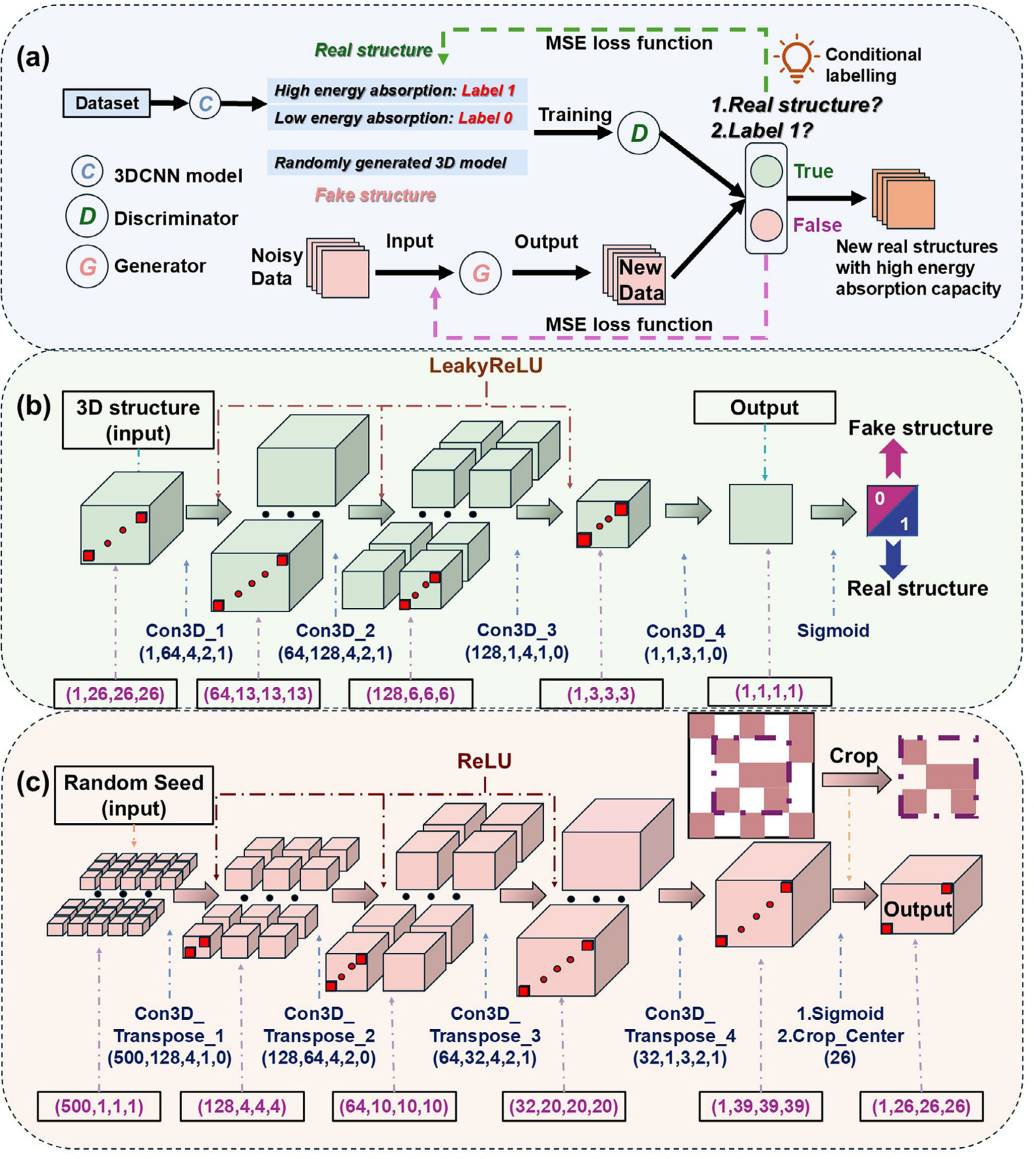
Schematic representation of (a) 3D conditional DCGAN framework combined with 3D CNN model, featuring: (b) Generator structure and (c) Discriminator structure.

Based on well‐trained CNN model, DCGAN [40] is then employed, which generally consists of a generator for generating new lattice designs and a discriminator for evaluating the rationality of the generated models. As shown in Figure 3a, to pre‐train the discriminator, the established 20 000 3D Gaussian mixture models included in the dataset were treated as real structures, while randomly generated voxel block combinations were regarded as noise during the training process. As for the “seed” configuration of the generator, an excessively low dimensionality might lead to overly similar input seeds for the generator in each iteration, whereas an excessively high dimensionality could make model convergence difficult and might result in overly sparse gradient signals in the space. Both scenarios would ultimately cause the structural models generated by the generator to lack diversity, a phenomenon known as “mode collapse”. Therefore, after multiple trials, we set the input seed as a randomly generated matrix composed of 50 values, which optimally balances the diversity of random seeds and the computational load on the generator. However, the traditional DCGAN only captures the geometric features of the models during training process and would not distinguish between the underlying categories of the models. Therefore, to achieve the goal of this work – generating novel structures with high energy absorption performance – we introduce label information into the discriminator's real‐structure input to distinguish between high and low energy‐absorbing structures (as mentioned in the previous section). This enables the discriminator to learn the geometric characteristics of the two distinct structural types. As shown in Figure 3a, we expand the input data by adding a new dimension containing only binary labels: “1” for high energy‐absorption structures and “0” for low energy‐absorption structures. At the generator stage, we constrain the generator's target output to be 3D lattice models labeled as “1”. The detailed structures of the generator and discriminator are presented in Figure 3b,c, respectively. For the generator component, the random seed undergoes a series of four 3D convolutional layers, ultimately yielding a novel 3D structure with dimensions of (39, 39, 39). In the present work, we introduce a structural center cropper module to precisely truncate the output, ensuring strict compliance with the required (26, 26, 26) dimensional specifications. To maintain non‐negative coordinate values throughout the generation process, ReLU (Rectified Linear Unit) activation functions are systematically employed between all convolutional layers. The discriminator architecture mirrors the 3D CNN structure described in the previous section, with significant enhancements to improve discrimination accuracy. Specifically, the convolutional layer stack has been expanded to four layers, matching the generator's depth for balanced adversarial training. The final classification of generated structures is accomplished through a sigmoid activation function, which provides probabilistic discrimination between ‘real’ and ‘fake’ samples.

**FIGURE 4:**
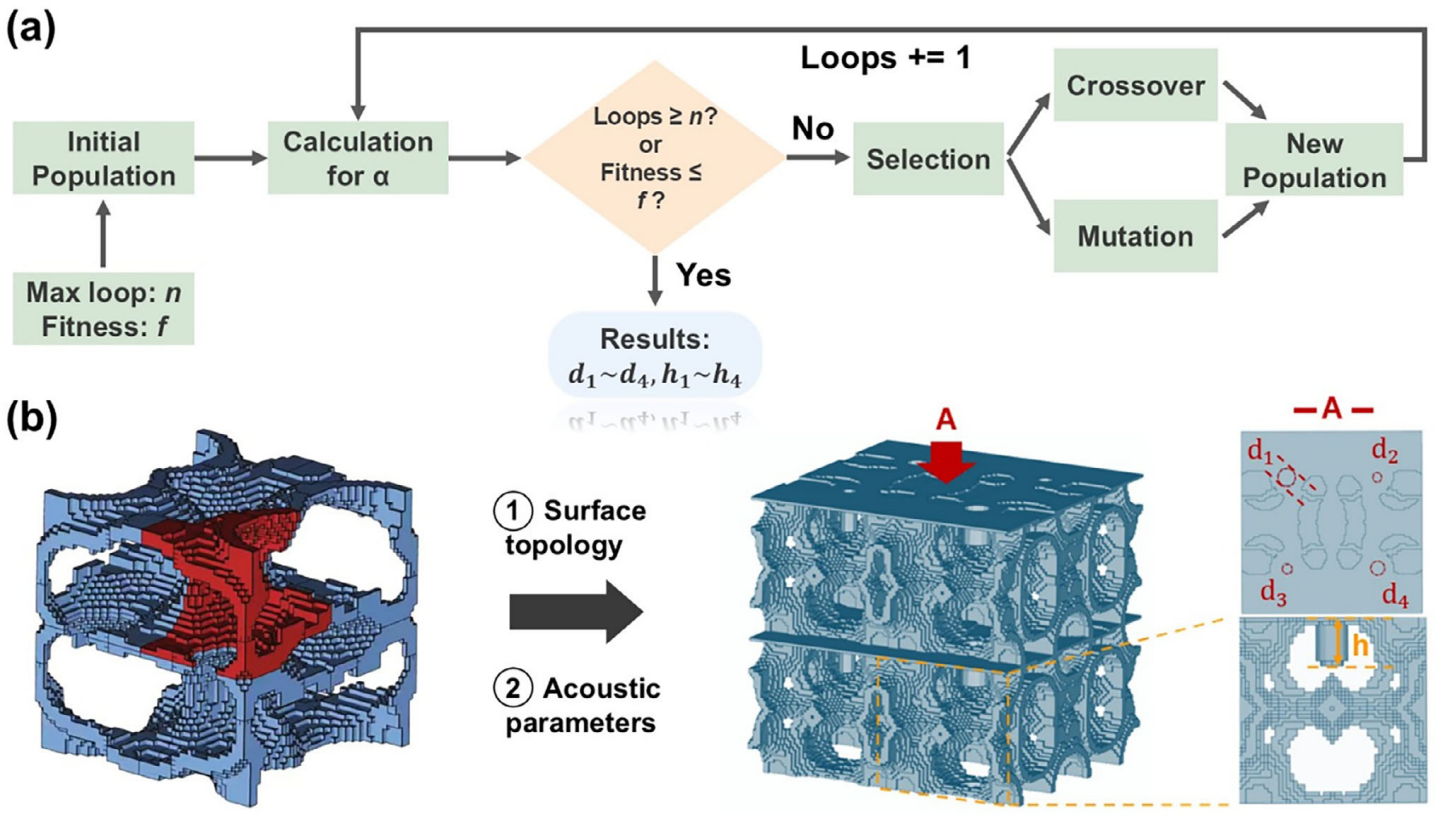
Schematic of the workflow for the introduction of acoustic geometries. (a) The workflow of genetic algorithm in determining the parameters of the diameter of the sound‐absorbing hole (*d*) and the length of the sound‐absorbing walls (*h*), and (b) the method in introducing optimized sets of acoustic geometries.

The acoustic performances of generated high‐energy absorption 3D structures by GAN are then optimized through the combination of governing equations and genetic algorithm as shown in Figure 4a. Due to its strong structure‐resonance coupling, Helmholtz resonance serves as the dominant mechanism for enabling high acoustic absorption in lattice materials [16]. Thus, it is imperative to introduce Helmholtz resonance to the metamaterial to allow effective sound absorption. Many prior studies have employed traditional surrogate models or surrogate models coupled with genetic algorithms to achieve inverse design for parametric optimization objectives [41, 42]. Our previous work utilized a similar hybrid approach, combining genetic algorithms and artificial neural networks to realize inverse design for superior acoustic performance in shell lattices [9]. However, the primary shortcoming of traditional surrogate‐based methods lies in their poor interpretability, which often rely on extensive datasets, and they are better suited for optimizing objectives such as non‐linear mechanical properties (such as mechanical energy absorption), which typically lack analytical solutions. In this study, the optimization process is grounded in the fundamental governing equations of Helmholtz resonators. This approach enhances the interpretability of the optimization procedure and involves a low‐dimensional parameter space. Consequently, the combination of a genetic algorithm with analytical solutions proves sufficient for accomplishing the inverse design task while significantly reducing optimization time and computational burden. In our structure, this is achieved by incorporating a pore of diameter *d*, and depth *h*, into each unit cell (Figure 4b). Since acoustic properties are size‐dependent, the unit cell is defined as a cube with an edge length of 5 mm. Then, along sound incidence, six unit cell layers are stacked in total. With extrinsic geometries fixed, consequently, *d* and *h* serve as the sole independent geometric parameters necessary to model and optimize sound absorption and are thus designated as the optimization targets in the genetic algorithm. To further enhance sound absorption, geometric heterogeneity is introduced by arranging four distinct unit cells in parallel. Details of the analytical acoustics calculations and optimization criteria can be found in Section S2. Overall, the independent variables *d* and *h*, in conjunction with other dependent parameters, form the basis for computing the acoustic impedance, which governs the resulting sound absorption coefficient spectrum of the lattice. Specifically, our objective is to identify optimal combinations of *d* and *h* across four heterogeneous unit cells, e.g., *d_1_* and *h_1_, … d_4_* and *h_4_*, with the highest average absorption coefficients (α) within the frequency range of 1000 to 5800 Hz (Figure 4b). The optimized acoustic parameters are listed in Table S1.

## 3 Results and Discussion

### 3.1 High Energy Absorption Capacity

**FIGURE 5:**
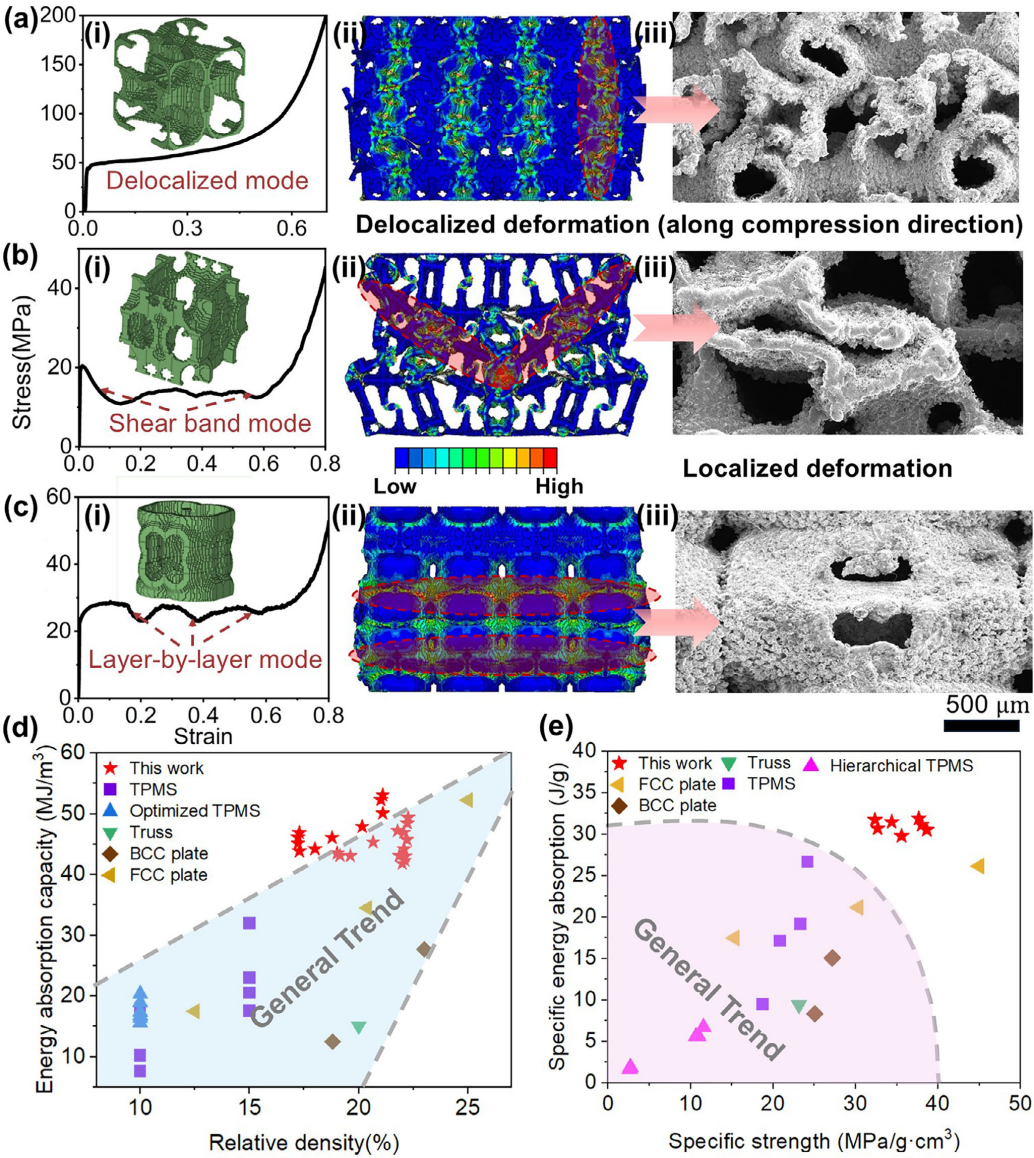
(a – c) (i) The strain–stress curves of three types of generated lattice structures with different compression behaviors: delocalized deformation, shear band formation, and layer by layer collapse. (ii) The strain contour plots, and (iii) SEM images of fracture surfaces. The comparison of (d) energy absorption capacity with respect to relative density, and (e) specific energy absorption with respect to specific strength, against structures reported in prior studies. (reported data: Optimized TPMS [9], Truss [44], BCC plate [45] and FCC plate [45]).

During the training process of the GAN model, when the discriminator and generator “co‐evolve” to improve accuracy, the errors of both models should stabilize around 1/2 – meaning the generator strives to produce reasonable structures that the discriminator cannot confidently classify as real or fake. Moreover, the loss function defined for the GAN model in this work is the MSE function. Due to the squared term in the error calculation, the loss value should theoretically stabilize around 0.25 during optimization [43]. As demonstrated in Figure S1, the experimental results confirm that our model exhibits strong generative capability and generalization performance. Regarding the deformation modes of the generated 3D lattice structures, they can be primarily categorized into delocalized deformation and localized deformation. As illustrated in Figure 5a‐i,ii, lattice structures exhibiting delocalized deformation characteristics demonstrate more uniform strain distribution during compression. This mechanical behavior is reflected in their stress–strain curves as relatively smooth profiles without abrupt fluctuations. In contrast, the localized deformation mode can be classified into two distinct mechanisms: shear band formation and layer‐by‐layer collapse. Upon entering the yield phase, the shear band exhibits a gradual reduction in strength, as illustrated in Figure 5b‐i,ii. Meanwhile, Figure 5c‐i,ii shows that each successive layer collapse triggers an abrupt drop in structural strength, indicating a layer‐by‐layer collapse mechanism. To distinguish the three deformation modes more clearly, the SEM images (Figure 5a–c‐iii) are utilized to show the corresponding deformed surfaces, respectively. Comparative analysis reveals that delocalized deformation behavior contributes significantly to enhancing the energy absorption capacity of lattice structures. Notably, the majority of high‐energy‐absorption structures generated in this study exhibit this favorable characteristic, as exemplified by the structure presented in Figure 5a‐i. This particular structure achieves remarkable mechanical performance, with a yield strength exceeding 50 MPa and demonstrating an outstanding energy absorption capacity of 55 MPa/m^3^ at a relative density below 19%.

Moreover, as illustrated in Figure 5d,e, the energy absorption performance of newly generated lattice structures by GAN model is compared against several state‐of‐the‐art designs, including high‐strength plate lattices and other topologically optimized architectures. Under identical relative density conditions, the novel structure proposed in this work demonstrates significantly enhanced energy absorption performance compared to conventional configurations. Specifically, it exhibits approximately 50%–100% greater energy absorption capacity than traditional plate structures, and a remarkable 200% improvement over truss structures. When evaluating specific energy absorption (SEA) metrics, the innovative architecture maintains superior performance while achieving the highest specific strength. Notably, its SEA values surpass those of TPMS structures by 40%–200%, establishing a new benchmark for lightweight energy‐absorbing materials. Additionally, compared to our previous lattice structures [9] optimized using implicit functions, this study not only improves energy absorption by up to 25% but also enhances structural design freedom, eliminating the pinch‐off issues caused by combining different implicit functions.

### 3.2 Elastic and Yielding Performance

**FIGURE 6:**
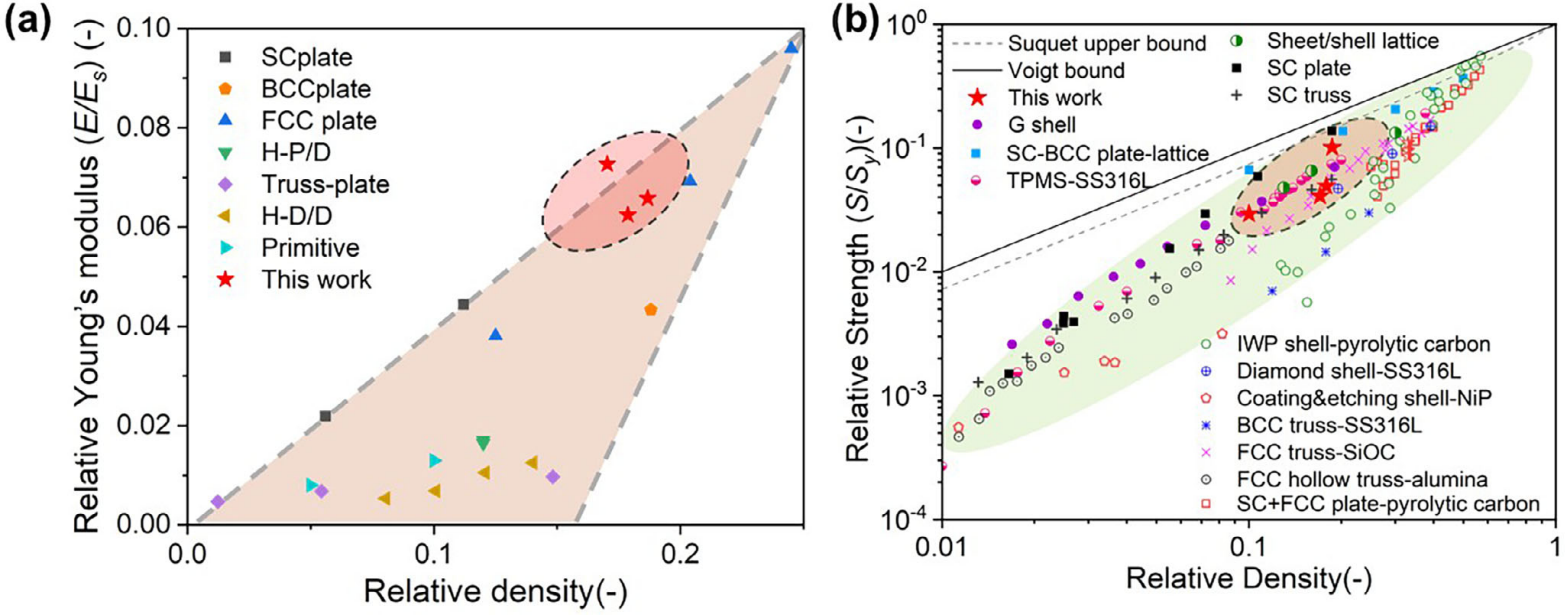
The comparison of mechanical performance between proposed lattice structures of this work and other typical lattice structures: (a) elastic modulus and (b) yield strength. (reported data: Hierarchical D‐D type with Boolean methods (H‐D/D) [48]; Hierarchical conformal P‐D type (H‐P/D) [49]; Single Gyroid shell (G shell) [48]; Single Primitive [48]; Hierarchical truss‐plate [50], SC plate, BCC plate and FCC plate [2]; SC truss [2]; SC + FCC plate‐pyrolytic carbon [51]; FJ‐SS316L [2]; SC‐BCC plate lattice [2]; IWP shell‐pyrolytic carbon [52]; Diamond shell‐SS316L [53]; Coating & etching shell‐NiP [54]; BCC truss‐SS316L [53]; FCC truss‐SiOC [55]; FCC hollow truss‐alumina [56]).

As shown in Figure S2 (Section S3), the geometric characteristics of proposed lattice are shown to possess the no self‐intersection and open‐cell characteristics. However, it is worth noticing that proposed lattice structures do not adhere to the constraints of cubic symmetry according to the projection views. Previous studies have also pointed out that the widespread applications of multifunctional lattice structures, which do not limit themselves to cubic symmetry [1, 46]. Then the elastic properties of the proposed high performance lattice structures in Figure 5a are shown in Table S2, where *K_eff_* and *a_ij_* represent the bulk modulus and Zener ratio, respectively. Then the elastic properties of the proposed high performance lattice structures in Figure 5a are shown in Table S2, where *K_eff_* and *a_ij_* represent the bulk modulus and Zener ratio, respectively. The results indicate that, as intended by the design, the proposed structures exhibit higher Young's modulus along the compressive zaxis compared to other directions, while still demonstrating favorable elastic performance in other orientations. For instance, Design 3 achieves a Young's modulus of 11.865 GPa along the yaxis under compression, even at a relatively low relative density of 17.87%. Then the elastic performances are compared with other typical lattice structures, such as TPMS, truss and plate lattices as shown in Figure 6a. The structure demonstrates that its Young's modulus performance can surpass that of most shell and truss structures, and even exhibits significant advantages over FCC and BCC plate structures, reaching a level comparable to that of SC plates. Considering the manufacturability issues associated with the closed‐cell geometric characteristics of SC plates, the structure proposed in this study achieves superior performance by balancing both high strength and high manufacturability.

The yield strength (σ_*Y*_) of the designed structure is also compared with other common lattice structures, as shown in Figure 6b. The primary metric employed was the yield strength under compressive plastic deformation along the (001) direction (Z‐axial in [100]‐unit). Results indicate that the lattice structures proposed herein outperforms most typical lattice configurations, exhibiting clear advantages over other designs such as truss‐based and shell‐based structures. It is noteworthy that SC plate structures and their variants display outstanding mechanical properties, approaching or even exceeding the Voigt and Suquet upper bounds. However, as documented in the literature, SC‐plate designs are hindered by notable manufacturability challenges and performance instability caused by micro‐holes. Subsequently, to comprehensively analyze the directional dependence of yield strength in the orthotropic lattice structure presented in this work, the compressive yield strengths of the high‐performance design model in Figure 5a along various crystallographic orientations [47] ([100]‐unit, [110]‐unit, and [111]‐unit) were evaluated, as summarized in Table S3. The [100]‐unit configuration demonstrates the most favorable performance, especially along the Z‐axis (52.51 MPa), which is approximately 2.5 times its X‐axial strength. The [110]‐unit shows the greatest anisotropy, with Z‐axial strength being over 5 times that of its weaker in‐plane (X, Y) directions. Although the [111]‐unit exhibits more balanced in‐plane strengths, its Z‐axial performance (17.98 MPa) is intermediate, falling between the [100] and [110]. This hierarchy highlights the direct link between crystal orientation and mechanical strength in the designed lattice. Then, the yield surface plots under principal plane stress condition are shown in Figure S3, which follows the governing equation (Equation S16). The yield strength with (001) direction in [100]‐unit, which represents the highest strength, is taken as an example to plot the yield surface.

### 3.3 High Sound Absorption Coefficients

**FIGURE 7:**
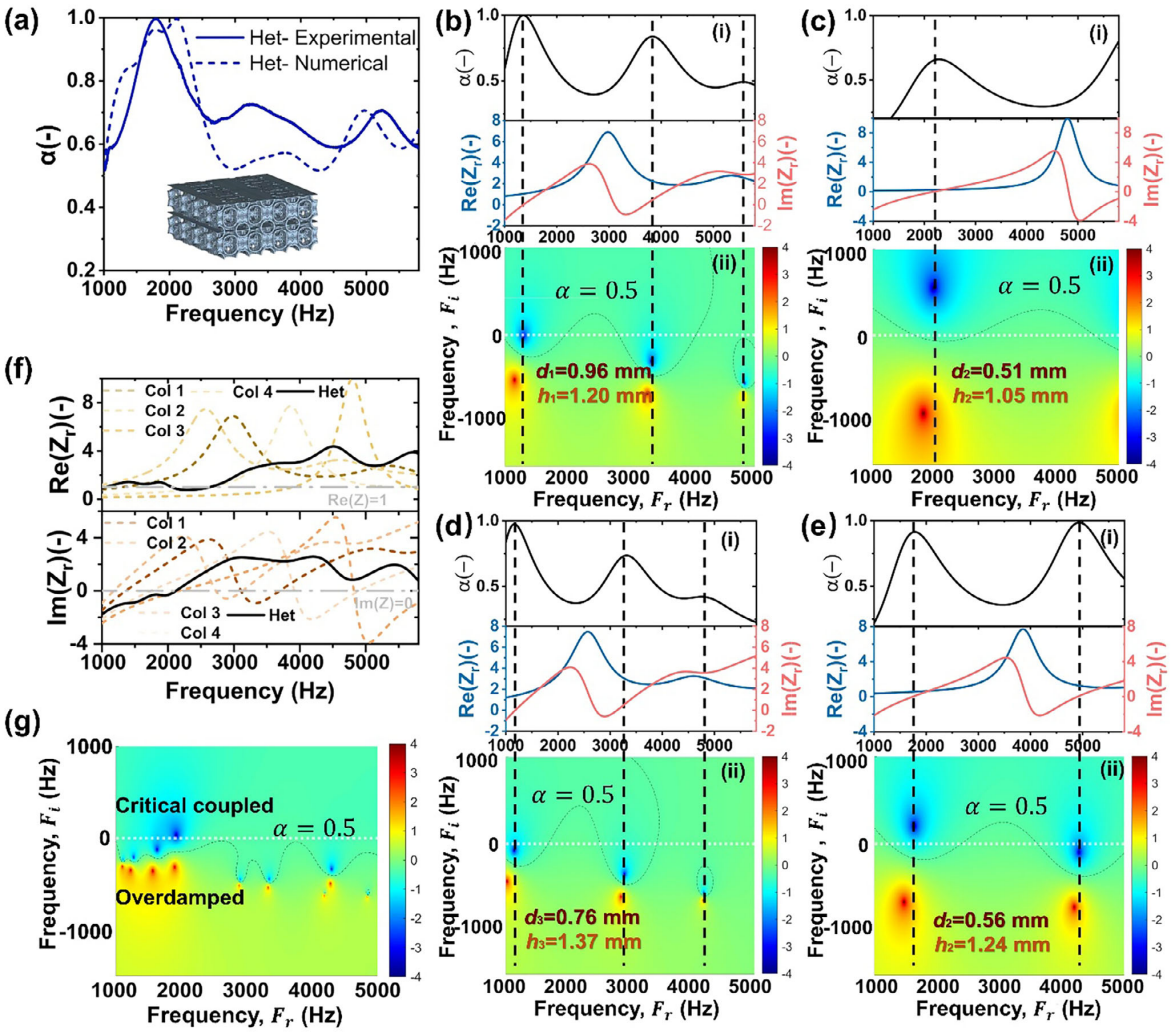
(a) The experimentally measured sound absorption coefficient curve of designed structure as compared with that of numerical model. (b‐e) (i) The absorption coefficient curves (α), the Re(Z_r_) and Im(Z_r_) portions of the acoustic impedance, and (ii) the complex planes of Four constitutive sound‐absorbing pores in heterogeneous structure. (f) The Re(Z_r_) and Im(Z_r_) curves of the acoustic impedance of heterogeneous structure, revealing curves closer to ideal values as compared to the constituent pores. (g) The complex plane of generated structure.

Experimental validation was performed using 3D‐printed lattice samples tested in an impedance tube. Indeed, Figure 7a displays a broadband sound absorption curve that is smooth and continuous across the frequency range, rather than comprising distinct peaks and valleys. Notably, the entire absorption spectrum remains above 0.5 with an average coefficient of 0.69, indicating effective sound absorption. This performance can be attributed to the synergistic behaviour of the four‐unit cells, whose resonance modes work collectively to enhance broadband attenuation. Additionally, from Figure 7a, a strong agreement between predicted values and experimental data can also be seen, demonstrating the high fidelity of our analytical methods. Comparing the sound absorption performance of our lattice design against several state‐of‐the‐art unit cells, including representative TPMS, plate, and truss lattices [9, 57], our design exhibits superior broadband absorption, maintaining consistently high values across the frequency range, in contrast to the narrowband peaks’ characteristic of the other structures. This broadband behaviour is particularly advantageous for practical applications, where wide frequency coverage is highly desirable. Furthermore, from an engineering design perspective, our lattice achieves this performance with a reduced sample thickness of just 30 mm, whereas the other designs require equal or greater thicknesses to deliver comparable results.

To further better understand the coupling mechanisms contributing to the enhanced broadband absorption, we delve into the impedance profiles of the metamaterial. The specific acoustic impedance (Z_r_) profiles of the optimized metamaterials, and its four constituent phases, are presented in Figure 7b–e‐i, respectively. The specific acoustic impedance is further decomposed into its real (Re(Z_r_)) and imaginary (Im(Z_r_)) components. Perfect sound absorption (i.e., α = 1) occurs when Re(Z_r_) = 1 and Im(Z_r_) = 0. In simple terms, a real part of 1 means the structure absorbs sound as effectively as air transmits it, while an imaginary part of 0 indicates no reactive resistance, together ensuring zero reflection and complete energy absorption. This condition is clearly observed at Figure 7f, where both Re(Z_r_) and Im(Z_r_) intersect the y‐axis at 1 and 0, respectively. Physically, the real part of the impedance corresponds to the amount of energy dissipated, while the imaginary part relates to the frequency range over which this dissipation occurs.

To visualize energy dissipation mechanisms, the complex plane plots of the reflection coefficient (R) are shown in Figure 7b–e‐ii. These plots use real (f_r_) and imaginary (f_i_) frequency components, with the colour map indicating the value of log_10_|R|^2^. The reflection coefficient R is related to absorption via α = 1 – |R|^2^. Resonant behaviour is revealed through pairs of conjugate poles (marked in red) and zeros (marked in blue). The conjugate zeros, where log_10_|R|^2^ is minimized, identify frequencies of peak absorption. Their positions offer insight into dissipation characteristics: a zero located on the real frequency axis (f_i_ = 0) indicates critically coupled damping, while those offset above or below signify underdamped or overdamped conditions, respectively. The number of pole‐zero pairs corresponds to the number of distinct local resonances. Peaks where α approaches 1 imply critical damping, while lower peaks may result from over‐ or under‐damping. To bridge the complex plane analysis with the absorption coefficient curves, ellipsoidal boundaries are drawn around regions where α ≥ 0.5, marked with dotted lines. The intersection of these regions with the real frequency axis aligns with frequencies showing α ≥ 0.5 in the absorption plots. As expected, the constituent phases exhibit distinct and isolated resonance regions, reflected by separated conjugate pole‐zero pairs, confirming local rather than broadband absorption behaviour. In contrast, the optimized metamaterial shows a continuous sequence of pole‐zero pairs spanning a broad frequency range as shown in Figure 7g, corresponding to an extended region where α ≥ 0.5.

## 4 Further Discussion

**FIGURE 8:**
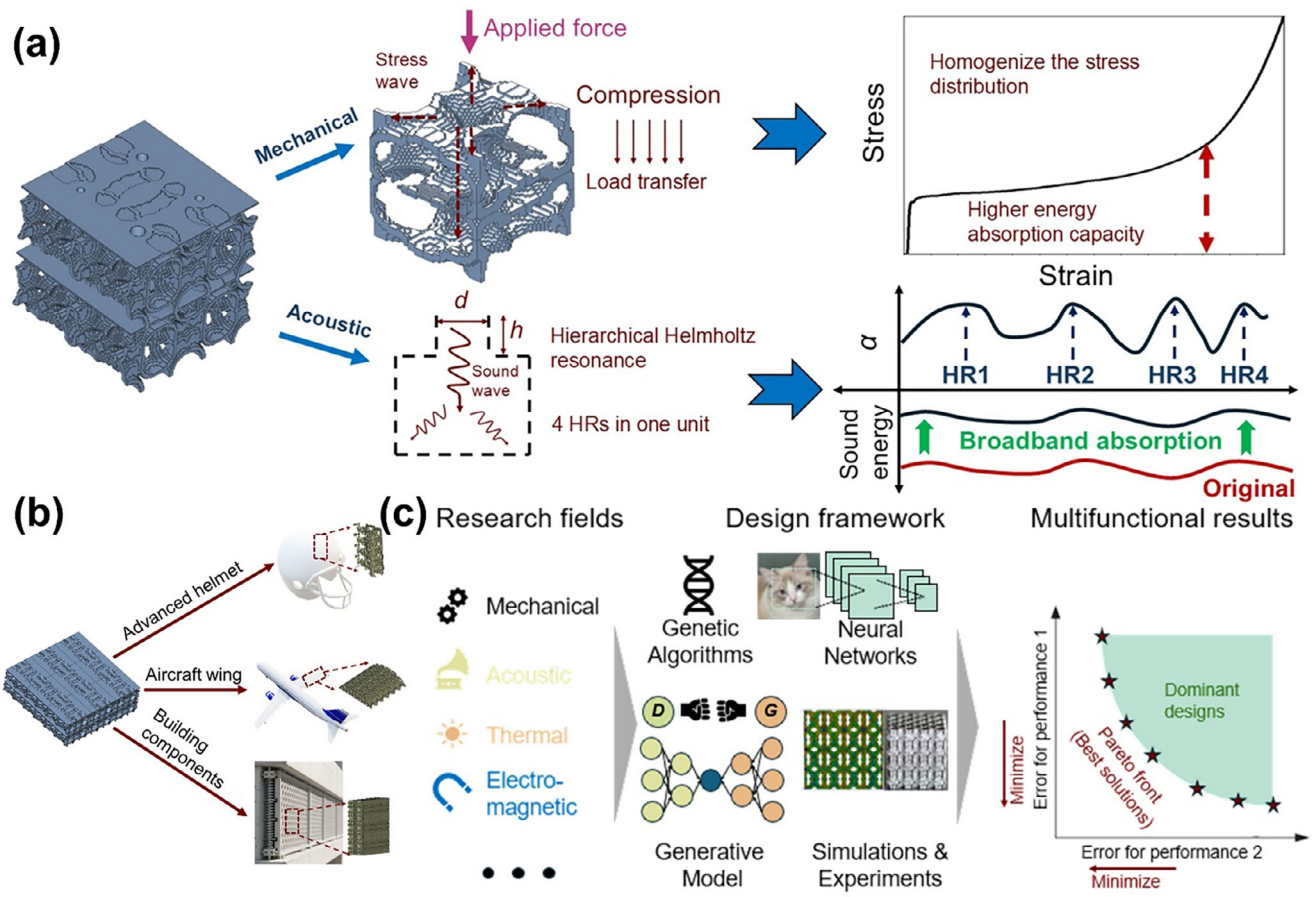
(a) The further explanation of mechanical and acoustic performances for designed metamaterials from the aspect of load transfer and hierarchical Helmholtz resonance. (b) The potential applications for acoustic‐mechanical coupling lattice metamaterials. (c) Illustration of the adaptability of the proposed framework to various research fields to design multifunctional structures with the best Pareto solutions.

This work presents a novel multifunctional lattice metamaterial optimization framework that integrates advanced methodologies including 3D DCGANs, CNNs, and GAs, achieving dual design objectives of high energy absorption and superior sound absorption performance. For energy‐absorbing metamaterials, the fundamental mechanism lies in enhancing stress distribution uniformity throughout the lattice structure. Through extensive computational training and iterative machine learning processes, our architecture develops the capability to “memorize” and replicate the characteristic structural patterns of high‐efficiency energy absorbers. As demonstrated in Figure 8a, the optimized lattice exhibits ideal deformation characteristics under compressive loading. The superior energy absorption capability stems from three key design innovations: First, the algorithm‐optimized unit cell geometry creates optimal stress distribution pathways that prevent localized stress concentrations. Second, self‐repeating architecture enables uniform stress wave propagation during impact events. Third, the machine learning model incorporates material plasticity parameters by FEM models during training, allowing the generated structures to balance stiffness requirements with controlled deformation behavior. Remarkably, despite these performance enhancements, our inverse‐designed lattice metamaterials maintain an ultralight relative density of approximately 20% through our optimization. The design employs mirror symmetrical constraints to ensure proper structural connectivity and manufacturability while eliminating unnecessary mass accumulation.

Regarding acoustic performance, the inherent porosity of lattice structures provides unique advantages for sound absorption applications [16]. Building upon previous work that employed ANN‐GAs for inverse design of shell lattices [9], this study advances the methodology by treating pore diameter (*d*) and depth (*h*) as coupled optimization parameters in a combinatorial acoustic design framework. As shown in Figure 8a, the multi‐objective optimization process simultaneously considers Helmholtz resonator (HR) effects from precisely tuned cavity dimensions and broadband impedance matching through graded porosity distribution. Our experimental results demonstrate that the optimized hierarchical HRs configuration achieves average absorption coefficients exceeding 0.5 across 1000–5800 Hz, representing a 25% improvement over conventional porous absorbers. Additional acoustic testing reveals that the graded pore arrangement effectively extends the working frequency band.

The proposed voxel‐Gaussian hybrid design enables lattice metamaterials with exceptional energy dissipation and broadband sound absorption, offering transformative applications across multiple industries. As shown in Figure 8b, human‐interface applications include advanced helmet designs that mitigate concussion risk through simultaneous energy absorption and sound attenuation. For civil infrastructure, earthquake‐resistant building components could simultaneously damp seismic waves and block urban noise. Industrial equipment benefits include targeted noise suppression in wind turbines and vibration isolation for precision instruments. Moreover, in aerospace and defense, such structures can enhance aircraft landing gear while reducing engine noise, and automotive applications include lightweight EV battery enclosures with integrated crash protection and noise suppression. The design's adaptability further permits active metamaterials incorporating shape‐memory alloys or piezo electrics for tunable performance. Coupled with additive manufacturing, these multifunctional lattices promise breakthroughs in sustainable infrastructure, next‐gen transportation, and advanced medical devices, where concurrent mechanical‐acoustic optimization is critical.

Meanwhile, this study introduces a novel framework for 3D lattice metamaterial design by integrating voxel blocks with multi‐layer 3D Gaussian distributions. Compared to conventional TO methods and implicit function‐based approaches, our architecture significantly expands design freedom, enabling exploration of previously unattainable performance boundaries. The voxel‐Gaussian hybrid model allows granular control over local porosity and anisotropic properties while maintaining global structural coherence. Furthermore, the incorporation of advanced 3D DCGANs enhances both geometric complexity and functional accuracy beyond traditional deep learning algorithms or rule‐based generative methods. The DCGANs' adversarial training process ensures high‐fidelity generation of microstructures with sub‐voxel feature resolution (<50µm), achieving an improvement in morphological diversity metrics. As shown in Figure 8c, beyond acoustic‐energy absorption structures, this hybrid methodology demonstrates extensibility to diverse multifunctional metamaterial. The initial functional properties such as thermal and electromagnetic performances can be obtained by experiments and FEM models. Then neural networks are employed to have accurate predictions. Subsequently, with the aids of GA and generative models, the design framework is dedicated to identifying the optimal solution set for multifunctional metamaterials, which is also called the Pareto front in multi‐objective optimization.

## 5 Conclusion

In conclusion, this study presents a general bottom‐up design methodology for achieving multifunctionality in architected lattice structures by integrating a novel Gaussian distribution‐based voxel generation approach with performance‐driven optimization. The concept is exemplified through the simultaneous enhancement of energy absorption and sound absorption. A hybrid deep learning framework—comprising a 3D convolutional neural network (CNN) for accurate energy absorption prediction (>98%) and a conditional DCGAN for inverse design—is coupled with genetic algorithm optimization of Helmholtz resonator parameters, enabling the creation of lattice architectures with both exceptional energy absorption and broadband acoustic attenuation (α > 0.5 across 1000–5800 Hz). The Gaussian voxel generation strategy represents a paradigm shift from traditional implicit function‐based methods, overcoming geometric limitations and significantly expanding the design space beyond TPMS‐like structures. Comparative analyses with numerical simulations and experimental validations show that the resulting designs outperform conventional lattices by 40%–200% and previously optimized structures by at least 25% in energy absorption. This generative AI‐driven framework not only addresses the efficiency and design space constraints of conventional topology optimization and machine learning methods but also offers a versatile foundation for future applications in thermal management, electromagnetic shielding, and biomedical engineering.

## 6 Methods

### 6.1 Additive Manufacturing

The proposed lattice metamaterials are fabricated by our high precision laser powder bed fusion (HP‐LPBF) machine, equipped with a continuous‐wave fiber laser (wavelength in 1070nm) featuring a fine beam spot size of 25 µm to achieve the high‐precision fabrication. The fabrication process involved the utilization of stainless steel 316L (SS316L) fine powder characterized by a particle size ranging from 5 to 25 µm. Such machine provided an ideal platform to fabricate structures with high resolution (minimum feature size <100 µm).

### 6.2 Uniaxial Compression

To rigorously validate the numerical models, experimental compression tests were performed using a servo‐hydraulic *MTS MODEL 370.10* testing machine, while the deformation process was simultaneously recorded using a high‐resolution *SONY α6400* digital camera system operating at 1 frame per two seconds (Figure S4). The comparative analysis between experimental measurements and simulation results, as presented in Figure S5, especially the corresponding strain‐stress curves (Figure S5d) demonstrated excellent agreement with correlation coefficients exceeding 0.95 across all tested configurations, thereby conclusively verifying the accuracy and reliability of the developed FEM framework. Building upon this validated computational approach, we systematically generated an extensive dataset comprising 1000 distinct numerical models encompassing a wide range of geometric parameters and topological configurations. For each model, the energy absorption capacity was quantitatively evaluated through numerical integration of the complete stress–strain response. This comprehensive dataset, incorporating both the structural geometries and their corresponding mechanical performance metrics, serves as the fundamental training database for developing a CNN capable of accurately predicting the energy absorption characteristics of novel architected materials without requiring additional time‐consuming finite element simulations or physical experiments.

### 6.3 Finite Element Method

The FEM is systematically employed to simulate the quasi‐static compression behavior of the newly generated architected structures, as illustrated in Figure S51a–c. The numerical simulations are conducted using the commercial finite element analysis software Abaqus/Explicit, where each computational model is constrained between two rigid plates – with the bottom plate fully fixed in all degrees of freedom and the top plate applying controlled compressive displacement at a constant engineering strain rate of 10^−^^3^ s^−^^1^ to obtain complete stress–strain responses. The material constitutive model implemented in these simulations is carefully derived from comprehensive uniaxial tensile tests performed on standard dogbone specimens with a uniform thickness of 1.5 mm, ensuring accurate representation of the base material's mechanical properties. The investigated 4  ×  4  ×  4 periodic lattice structures are discretized into finite element meshes using eight‐node linear brick (C3D8) elements, with each cubic unit cell precisely dimensioned at 10  ×  10  ×  10 mm after conducting mesh sensitivity analysis to guarantee solution convergence while maintaining computational efficiency.

### 6.4 Boundary Conditions

For the evaluation of elastic and yield performances of generated lattice structure, the FEM under uniaxial strain loading and pure shear loading is implemented, integrated with the representative unit element. Periodic boundary conditions are first applied to the node pairs at opposite end faces [58]:

(7) $$u_{i}^{+}-\;u_{i}^{-}=\varepsilon_{ij}\;\left(X_{j}^{+}-X_{j}^{-}\right),\left(i=1,2,3\right)$$

where ‘+’ and ‘−’ represent node in the positive and negative directions, respectively. Besides, *j* is a dummy index of the Einstein summation convention and *u* is the translational vector. Then the boundary conditions imposed on the unit cell for the uniaxial strain loading ($\varepsilon^{\left(U\right)}={\left[\varepsilon_{11}^{\left(U\right)},0,0,0,0,0\right]}^{T}$) are:

(8) $$\begin{array}{ccc} u_{1}^{x+} & = & \varepsilon_{11}^{\left(U\right)}\;D,\;\;u_{1}^{y+}=0,\;\;u_{1}^{Z+}=0,\;u_{1}^{x+}=0,\;\;u_{1}^{y+}=0,\;\;u_{1}^{Z+}=0,\\ & & \;u_{1}^{x+}=0,\;\;u_{1}^{y+}=0,\;\;u_{1}^{Z+}=0 \end{array}$$

where *D* represents the length of cell size for unit‐cell. After that, the pure shear loading ($\varepsilon^{\left(U\right)}={\left[0,0,0,0,0,2\varepsilon_{12}^{\left(U\right)}\right]}^{T}$) are:

(9) $$\begin{array}{ccc} u_{1}^{x+} & = & 0,\;\;u_{1}^{y+}=\varepsilon_{11}^{\left(U\right)}\;\frac{D}{2},\;\;u_{1}^{Z+}=0,u_{1}^{x+}=\varepsilon_{11}^{\left(U\right)}\;\frac{D}{2},\;\;u_{1}^{y+}=0,\\ & & \;\;u_{1}^{Z+}=0,\;u_{1}^{x+}=0,\;\;u_{1}^{y+}=0,\;\;u_{1}^{Z+}=0 \end{array}$$

### 6.5 Sound Absorption Test

The sound absorption coefficient of the 3D printed lattice samples was experimentally measured using a BSWA SW4601 impedance tube, in accordance with ASTM E1050‐19 standards. The sample holder tube featured a 29 mm diameter opening, with the lattice samples designed to snug fit into the holder. Measurements were conducted over a frequency range of interest from 1000 to 5800 Hz.

## Funding

The authors would like to acknowledge the financial support of the Hong Kong Special Administrative Region (HKSAR) University Grants Committee – General Research Fund CUHK14209523 and CUHK14206925, the Innovation and Technology Commission – Innovation and Technology Fund ITP/058/23TP, as well as the Singapore Ministry of Education Academic Research Fund Tier 1 Grant (Grant No. A‐8002418‐00‐00).

## Conflicts of Interest

The authors declare no conflicts of interest.

## Supporting information




**Supporting File**: advs74594‐sup‐0001‐SuppMat.docx.

## Data Availability

The data that support the findings of this study are available from the corresponding author upon reasonable request.
