# Supplementary material for: A Bottom‐Up Design Framework for Multifunctional Lattice Metamaterials
Source: Adv Sci (Weinh). 2026 Feb 26;13(26):e18923. doi: 10.1002/advs.202518923 (PMC13159119; doi:10.1002/advs.202518923)
Supplement: Supplementary file 1 — Supporting File: advs74594‐sup‐0001‐SuppMat.docx. [file ADVS-13-e18923-s001.docx]

**A Bottom-Up Design Framework for Multifunctional Lattice Metamaterials**

Zongxin Hu ^a^, Quanqing Tao ^a^, Junhao Ding ^a^, Shuo Qu ^a^, Haitao Ye ^a^, Jun Wei Chua ^b^, Tianxiao Niu ^a^, Rui Li ^a^, Winston Wai Shing Ma ^a^, Haoming Mo ^a^, Hui Liu ^a^, Wei Zhai ^b, *^, Xinwei Li ^c, *^ Xu Song ^a, *^

^a^ *Department of Mechanical and Automation Engineering, Chinese University of Hong Kong, Shatin, Hong Kong SAR*

^b^ *Department of Mechanical Engineering, National University of Singapore (NUS), Singapore*

^c^ *Newcastle University in Singapore, Faculty of Science, Agriculture, and Engineering, Newcastle University, Newcastle upon Tyne NE1 7RU, UK*

Section S1: SEM images for printing quality


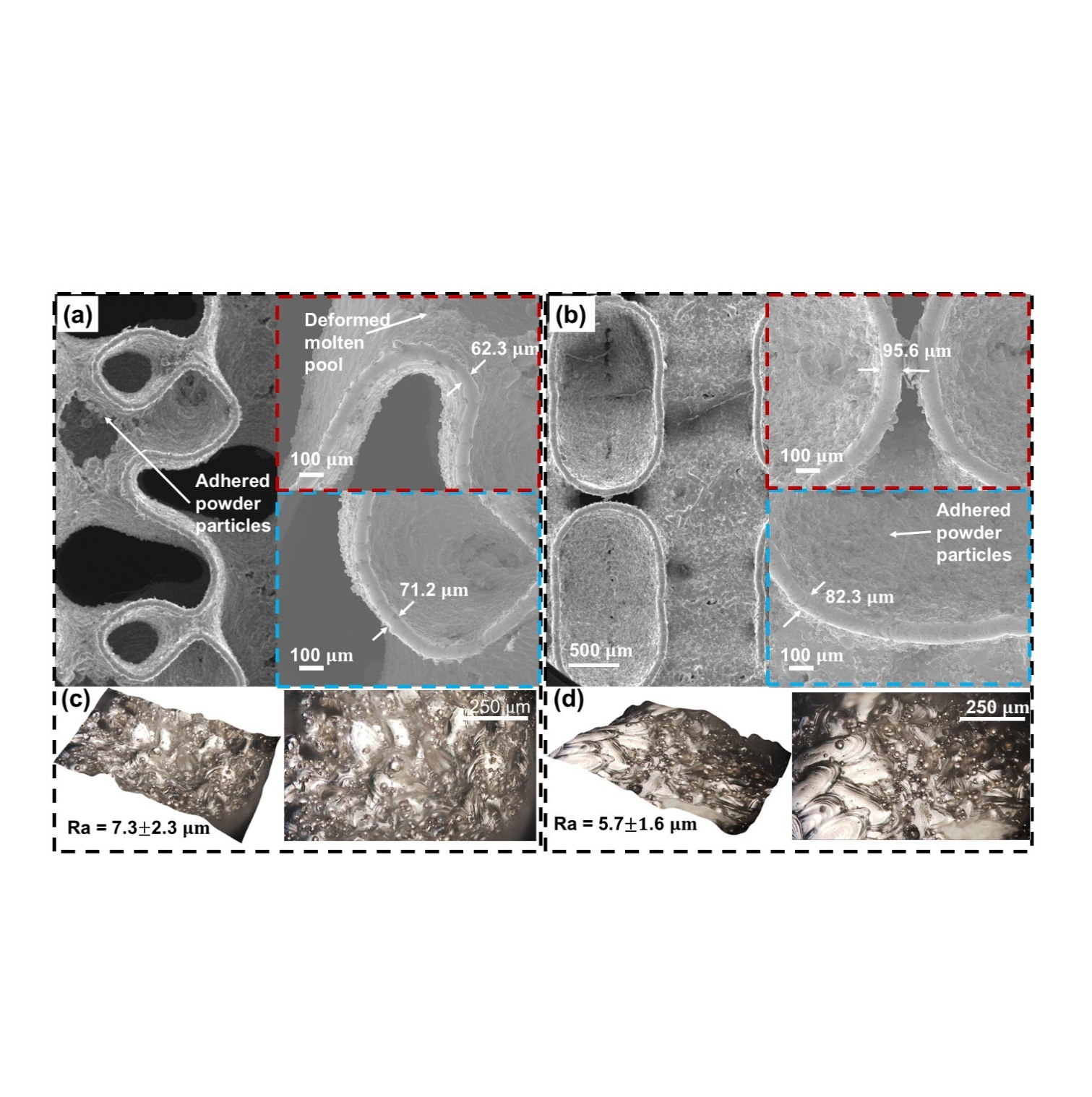


Fig. S1 (a) and (b) The dimensional accuracy and geometric defects of the printed samples. (c) and (d) The surface roughness of the printed samples

**Section S2 The optimization process of sound absorption coefficients**

Analytical models are adopted to calculate the sound absorption coefficient curves. For our design, we introduce pores to enable sound dissipation mechanisms through the multi-layered Helmholtz resonance mechanism. Thus, we employ the transfer matrix method (TMM) to model the equivalent-fluid flow across the pores and the cavities.^[1]^ TMM relates the acoustic pressure (*P*) and normal velocity (*v_x,in_*) at the inlet (x = 0) and outlet (x = L) of a sound-absorbing material backed by a rigid wall, as expressed in the following equation:

$$\begin{aligned} \left[ \begin{matrix} P_{in} \\ v_{x,in} \end{matrix} \right]_{x=0}=T\left[ \begin{matrix} P_{out} \\ 0 \end{matrix} \right]_{x=L}\#\left( S1 \right) \end{aligned}$$

T represents the 2×2 transfer matrix corresponding to the sound-absorbing material. The expressions for its four components are highly dependent on the metamaterial architecture and take on different forms based on its specific configuration.

In the case of multi-layer Helmholtz resonator, there are two transfer matrices related: *T_P_* for the narrow pore, and *T_C_* for the cavity. *T_P_* is related to the acoustic impedance of the pore, *Z_P_*, dependent on acoustical geometrical parameters such as the pore diameter (*d*), pore height (*h*), size of a fundamental unit (*W*), and the surface porosity (*ϕ*). Herein, *ϕ* is calculated via $\pi{(d/2)}^{2}/W^{2}$. T_C_ is in turn related to the cavity geometry, specifically the cavity depth, *D*, given as *W − h*. It is to note that W corresponds to 5 mm in our design here.

We first look at the acoustic impedance of the pore, *Z_P_*:

$$\begin{aligned} Z_{P}=\frac{1}{\phi}\left( i\omega\rho_{e}t+2\varepsilon R_{s}+i\omega\rho_{0}\delta d \right)\#\left( S2 \right) \end{aligned}$$

The first term in the bracket corresponds to the total acoustic impedance inside a pore. It depends on the effective airflow density, *ρ_e_*, and is determined using the following expression:

$$\begin{aligned} \rho_{e}=\rho_{0}\left( 1+\frac{\sigma\phi}{i\omega\rho_{0}}G_{c} \right)\#\left( S6 \right) \end{aligned}$$

*ρ_₀_* denotes the standard air density, while ω represents the angular frequency. The term *σ* corresponds to the airflow resistivity, which is influenced by the pore geometry and hydraulic radius. In the case of a circular pore, *σ* is given by ^[2]^:

$$\begin{aligned} \sigma=\frac{8\eta}{\phi\left( \frac{d}{2} \right)^{2}}\#\left( S3 \right) \end{aligned}$$

η denotes the dynamic viscosity of air, while G_c_ is defined in relation to the characteristic viscous length, s, as:

$$\begin{aligned} G_{c}={-\frac{s}{4}\sqrt{-i}\frac{J_{1}\left( s\sqrt{-i} \right)}{J_{0}(s\sqrt{-i)}}}/\left[ 1-\frac{2}{s\sqrt{-i}}\frac{J_{1}\left( s\sqrt{-i} \right)}{J_{0}(s\sqrt{-i)}} \right]\#\left( S4 \right) \end{aligned}$$

$$\begin{aligned} s=C\sqrt{\frac{8\omega\rho_{0}}{\sigma\phi}}\#\left( S5 \right) \end{aligned}$$

*C* is a shape dependent factor given as $C={\sqrt{{8\eta}/{(\sigma\phi)}}}/r$ for a circular pore. J_1_ and J_0_ are the first and zeroth order Bessel functions, respectively.

For the second term in Equation (S2), *R_s_* is an expression given by $R_{s}={\sqrt{2\eta\rho_{0}\omega}}/2$. Additionally, the second and third terms also account for the end correction factors associated with resistance (*ε*) and mass reactance (*δ*), respectively, which arise from airflow through a narrow pore. These correction factors are essential for capturing the acoustic interactions that extend beyond the pore’s immediate vicinity. Based on our previous study involving similar acoustic geometries,^[3]^ the best-fit values of *ε* and *δ*, corresponding to various pore diameters and thicknesses (in millimetres), are provided as follows:

$$\begin{aligned} \delta= \left( 0.3d - 0.05 \right) \#\left( S6 \right) \end{aligned}$$

$$\begin{aligned} \varepsilon= -6d + \left( \frac{10}{3} \right)t +\frac{37}{6}\#\left( S7 \right) \end{aligned}$$

Following this, *Z_P_* is fully characterized. The transfer matrix of the pore (*T_P_*) is then formulatd as:

$$\begin{aligned} T_{P}=\left[ \begin{matrix} 1 & Z_{P} \\ 0 & 1 \end{matrix} \right]\#\left( S8 \right) \end{aligned}$$

In contrast to the pore, the acoustical behavior of the cavity is only governed by its depth, *D*. The corresponding transfer matrix of the cavity, *T_C_*, is expressed as:

$$\begin{aligned} T_{C}=\left[ \begin{matrix} \cos\left( k_{0}D \right) & iZ_{0}\sin\left( k_{0}D \right) \\ \frac{i\sin\left( k_{0}D \right)}{Z_{0}} & \cos\left( k_{0}D \right) \end{matrix} \right]\#\left( S9 \right) \end{aligned}$$

Here, k_0_ denotes the wavenumber, and *Z_0_ = ρ_0_c_0_* represents the acoustic impedance of air under standard conditions, where *c_0_* is the speed of sound in air at those conditions. The overall transfer matrix for our lattice metamaterial comprises 6 alternating layers of pores and cavities is given by:

$$\begin{aligned} T_{T}=\prod_{i=1}^{6} T_{i}=T_{P1}\cdot T_{C1}\cdot T_{P2}\cdot T_{C2}\cdots T_{P6}\cdot T_{C6}=\left[ \begin{matrix} T_{11} & T_{12} \\ T_{21} & T_{22} \end{matrix} \right]\#\left( S10 \right) \end{aligned}$$

The relative acoustic impedance, *Z_r_*, can then derived from *T_T_* through the following transformation:

$$\begin{aligned} Z_{r}=\frac{{T_{11}}/{T_{21}}}{Z_{0}}\#\left( S11 \right) \end{aligned}$$

The sound absorption coefficient, α, at each frequency, is calculated using the following expression:

$$\begin{aligned} \alpha=\frac{4Re(Z_{r})}{[1+Re\left( Z_{r} \right)]^{2}+Im(Z_{r})^{2}}\#\left( S12 \right) \end{aligned}$$

The sound absorption coefficient curve across a broad spectrum is then obtained by plotting out α at each frequency interval.

When resonant cells with different acoustical geometries, characterized by the independent variables *d* and *h* in our case, are arranged perpendicular to the direction of sound incidence and treated as partitioned units, the resulting absorption coefficient curve typically represents a superposition of the individual responses. To compute the absorption coefficients of such heterogeneous assemblies, the parallel addition rule is applied. For each unit cell, the characteristic specific acoustic impedance *Z_r,i_* is calculated by substituting the corresponding values of d and h into the transfer matrix formulations previously defined in Equations (S2) through (S13). To derive the overall acoustic impedance (*Z_Het_*) of the assembled cell, the parallel rule addition is applied across all *Z_r,i_:*

$$\begin{aligned} Z_{Het}=\frac{A}{\sum_{i=1}^{n} \frac{A_{i}}{Z_{r,i}}} \#\left( S13 \right) \end{aligned}$$

where n represents the total number of cells, A_i_ denotes the normal surface area of each individual cell, and A the total normal surface area of the assembly. The sound absorption coefficients of the heterogeneous absorber are then calculated in a similar way using equation (S16). To obtain the optimized structure, a genetic algorithm is employed to identify the combination of four sets of acoustic geometries, *d_1_, h_4_ … d_4_, h_4_,* that yields the highest average absorption coefficient across the 1000 to 5800 Hz frequency range.

Table. S1. The optimized parameters for acoustic perforamnce by genetic algorithm

| Pore_model | 1 | 2 | 3 | 4 |
| --- | --- | --- | --- | --- |
| *d (mm)* | 0.96 | 0.51 | 0.76 | 0.56 |
| *h (mm)* | 1.20 | 1.05 | 1.37 | 1.24 |

Section S3: The geometric characteristics of proposed lattice


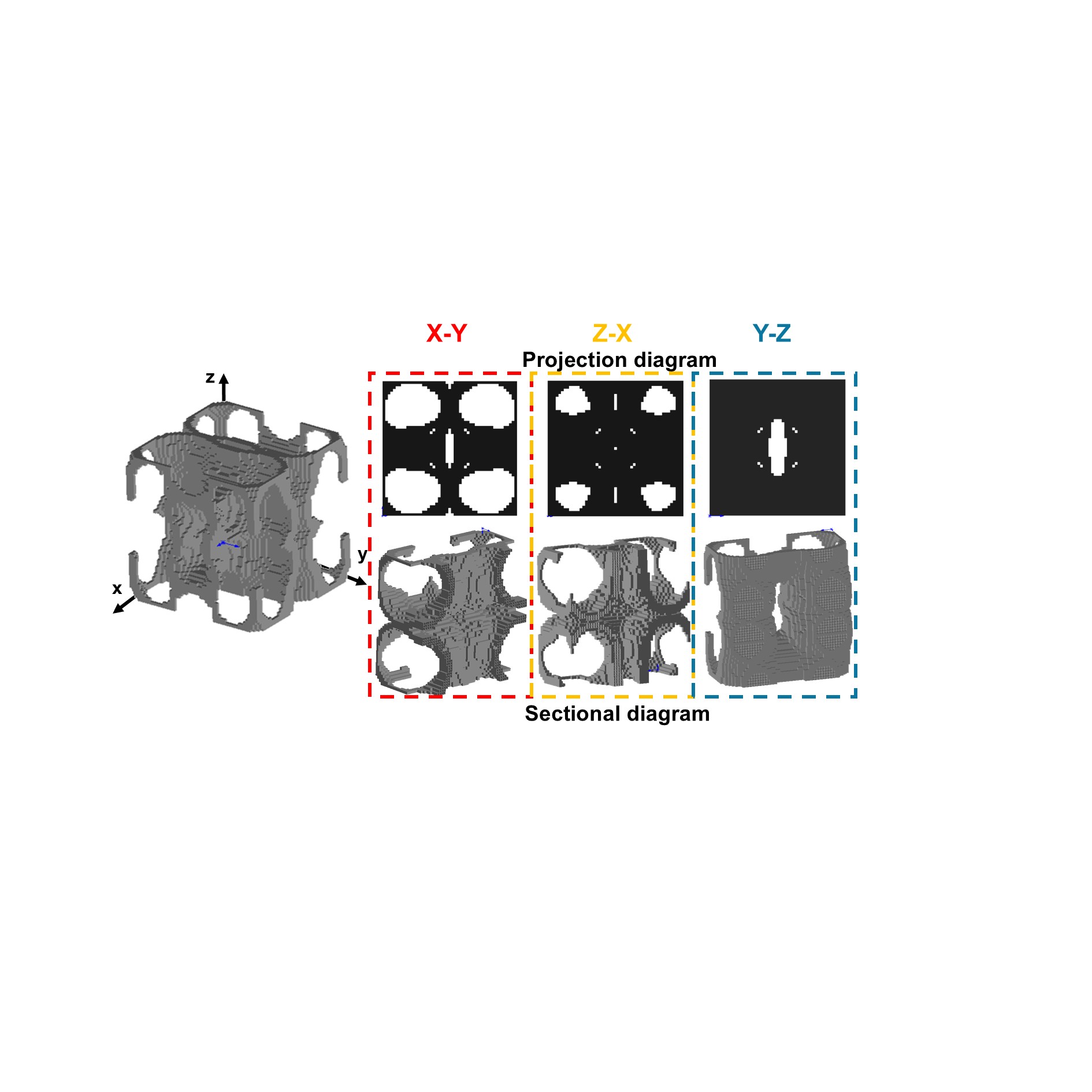


Fig. S2 The projectional and sectional diagrams of generated lattice structures

Section S4 : Elastic properties

Based on Hooke’s law, the elastic stiffness matrix for orthotropic materials in this work is determined as:

$$\begin{aligned} \left[ \begin{matrix} \sigma_{xx} \\ \sigma_{yy} \\ \begin{matrix} \sigma_{zz} \\ \sigma_{yz} \\ \begin{matrix} \sigma_{zx} \\ \sigma_{xy} \end{matrix} \end{matrix} \end{matrix} \right]=\left[ \begin{matrix} \begin{matrix} \begin{matrix} C_{11} \\ \begin{matrix} C_{12} \\ C_{13} \\ \begin{matrix} 0 \\ \begin{matrix} 0 \\ 0 \end{matrix} \end{matrix} \end{matrix} \end{matrix} & \begin{matrix} C_{12} \\ \begin{matrix} C_{22} \\ C_{23} \\ \begin{matrix} 0 \\ 0 \\ 0 \end{matrix} \end{matrix} \end{matrix} \end{matrix} & \begin{matrix} \begin{matrix} \begin{matrix} C_{13} \\ \begin{matrix} C_{23} \\ C_{33} \\ \begin{matrix} 0 \\ 0 \\ 0 \end{matrix} \end{matrix} \end{matrix} & \begin{matrix} 0 \\ \begin{matrix} 0 \\ 0 \\ \begin{matrix} C_{44} \\ 0 \\ 0 \end{matrix} \end{matrix} \end{matrix} \end{matrix} & \begin{matrix} \begin{matrix} 0 \\ \begin{matrix} 0 \\ 0 \\ \begin{matrix} 0 \\ C_{55} \\ 0 \end{matrix} \end{matrix} \end{matrix} & \begin{matrix} 0 \\ \begin{matrix} 0 \\ 0 \\ \begin{matrix} 0 \\ 0 \\ C_{66} \end{matrix} \end{matrix} \end{matrix} \end{matrix} \end{matrix} \end{matrix} \right]\left[ \begin{matrix} \varepsilon_{xx} \\ \varepsilon_{yy} \\ \begin{matrix} \varepsilon_{zz} \\ {2\varepsilon}_{yz} \\ \begin{matrix} {2\varepsilon}_{zx} \\ {2\varepsilon}_{xy} \end{matrix} \end{matrix} \end{matrix} \right]\#\left( S14 \right) \end{aligned}$$

And the inverse of this relation can be written as:

$$\begin{aligned} \left[ \begin{matrix} \varepsilon_{xx} \\ \varepsilon_{yy} \\ \begin{matrix} \varepsilon_{zz} \\ {2\varepsilon}_{yz} \\ \begin{matrix} {2\varepsilon}_{zx} \\ {2\varepsilon}_{xy} \end{matrix} \end{matrix} \end{matrix} \right]=\left[ \begin{matrix} \begin{matrix} \begin{matrix} 1/E_{x} \\ \begin{matrix} -\upsilon_{xy}/E_{x} \\ -\upsilon_{xz}/E_{x} \\ \begin{matrix} 0 \\ \begin{matrix} 0 \\ 0 \end{matrix} \end{matrix} \end{matrix} \end{matrix} & \begin{matrix} -\upsilon_{yx}/E_{y} \\ \begin{matrix} 1/E_{y} \\ -\upsilon_{yz}/E_{y} \\ \begin{matrix} 0 \\ 0 \\ 0 \end{matrix} \end{matrix} \end{matrix} \end{matrix} & \begin{matrix} \begin{matrix} \begin{matrix} {-\upsilon}_{zx}/E_{z} \\ \begin{matrix} -\upsilon_{zy}/E_{z} \\ 1/E_{z} \\ \begin{matrix} 0 \\ 0 \\ 0 \end{matrix} \end{matrix} \end{matrix} & \begin{matrix} 0 \\ \begin{matrix} 0 \\ 0 \\ \begin{matrix} 1/G_{yz} \\ 0 \\ 0 \end{matrix} \end{matrix} \end{matrix} \end{matrix} & \begin{matrix} \begin{matrix} 0 \\ \begin{matrix} 0 \\ 0 \\ \begin{matrix} 0 \\ 1/G_{zx} \\ 0 \end{matrix} \end{matrix} \end{matrix} & \begin{matrix} 0 \\ \begin{matrix} 0 \\ 0 \\ \begin{matrix} 0 \\ 0 \\ 1/G_{xy} \end{matrix} \end{matrix} \end{matrix} \end{matrix} \end{matrix} \end{matrix} \right]\left[ \begin{matrix} \sigma_{xx} \\ \sigma_{yy} \\ \begin{matrix} \sigma_{zz} \\ \sigma_{yz} \\ \begin{matrix} \sigma_{zx} \\ \sigma_{xy} \end{matrix} \end{matrix} \end{matrix} \right]\#\left( S15 \right) \end{aligned}$$

where $C_{11}$, $C_{12}$…$C_{66}$ are elastic constants which define the elastic properties of lattice structures. Besides, $E_{i}$ and $G_{ij}$ represent the Young’s modulus along axis *i* and the [shear modulus](https://en.wikipedia.org/wiki/Shear_modulus) within the plane spanned by axis *i* and axis *j*, respectively. $\upsilon_{ij}$ is the [Poisson's ratio](https://en.wikipedia.org/wiki/Poisson%27s_ratio) that corresponds to a contraction in direction *j* when an extension is applied in direction *i*. Then uniaxial strain loading and pure shear loading are adopted for evaluation of the elastic properties of the lattice. The details of boundary conditions and calculation procedures are shown in support information.

Table. S2 The elastic performance of three proposed representative lattice structures

| Elastic performance | Design_1 | Design_2 | Design_3 |
| --- | --- | --- | --- |
| $E_{x}$(GPa) | 4.014 | 1.753 | 1.655 |
| $E_{y}$(GPa) | 5.106 | 3.248 | 11.865 |
| $E_{z}$(GPa) | 12.498 | 13.809 | 12.294 |
| $G_{yz}$(GPa) | 7.948 | 7.802 | 6.320 |
| $G_{zx}$(GPa) | 2.673 | 1.386 | 0.296 |
| $G_{xy}$(GPa) | 0.341 | 0.248 | 0.529 |
| $\upsilon_{xy}$(-) | 0.471 | 0.872 | 0.209 |
| $\upsilon_{yx}$(-) | 0.369 | 0.470 | 0.028 |
| $\upsilon_{xz}$(-) | 0.429 | 0.103 | 0.298 |
| $\upsilon_{zx}$(-) | 0.138 | 0.013 | 0.042 |
| $\upsilon_{yz}$(-) | 0.095 | 0.430 | 0.256 |
| $\upsilon_{zy}$(-) | 0.039 | 0.101 | 0.265 |
| $K_{eff}$(GPa) | 25.494 | 2.350 | 8.604 |
| $a_{xy}$(-) | 0.251 | 2.037 | 0.902 |
| $a_{yz}$(-) | 3.399 | 4.943 | 1.293 |
| $a_{zx}$(-) | 1.915 | 1.767 | 0.559 |

**Section S5:** Plastic properties

Table. S3 The analysis of directional dependence of yield strength for proposed lattice model

| Direction | X-axial  (100)/ (110)/ (111) | Y-axial  (010)/ ($\bar{1}$10)/ (1$\bar{1}$0) | Z-axial  (001)/ ($001$)/ (11$\bar{2}$) |
| --- | --- | --- | --- |
| [100]-unit  (100), (010), (001) | 20.70 MPa | 23.89 MPa | 52.51MPa |
| [110]-unit  (110), ($\bar{1}$10), (001) | 6.52 MPa | 8.72MPa | 44.09MPa |
| [111]-unit  (111), (1$\bar{1}$0), (11$\bar{2}$) | 9.86 MPa | 7.02MPa | 17.98MPa |

The yield surface is the boundary of a closed domain in stress space that defines the elastic range of the material for pure elastic response in respective loading direction ^[4]^. The yield surface plots under principal plane stress condition follows the governing equation:

$$\begin{aligned} \sigma_{Y}=\sqrt{\frac{1}{2}\left[ {{(\sigma}_{xx}-\sigma_{yy})}^{2}+{{(\sigma}_{yy}-\sigma_{zz})}^{2}+{{(\sigma}_{zz}-\sigma_{xx})}^{2} \right]+3{(\sigma}_{xy}^{2}+\sigma_{yz}^{2}+\sigma_{zx}^{2})}\#\left( S16 \right) \end{aligned}$$

Then the lattice sample is subjected to the various stress states (biaxial plane stress, simple shear and various combinations of $\sigma_{xx}$, $\sigma_{xy}$) as shown in Fig. S3.


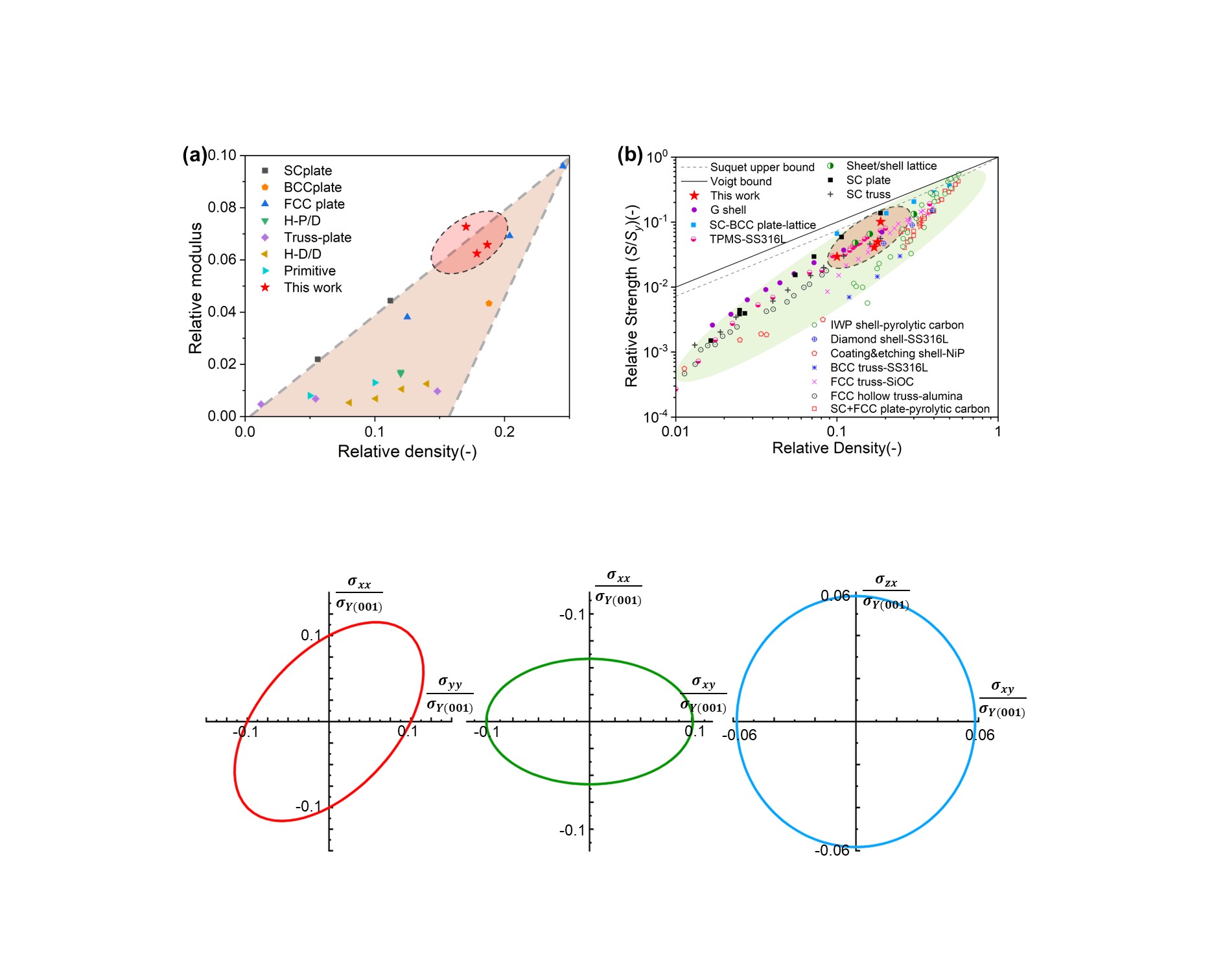


Fig. S3 The yield surface plots under principal plane stress conditions for lattice structures.

**Section S6:** Experimental and numerical methods


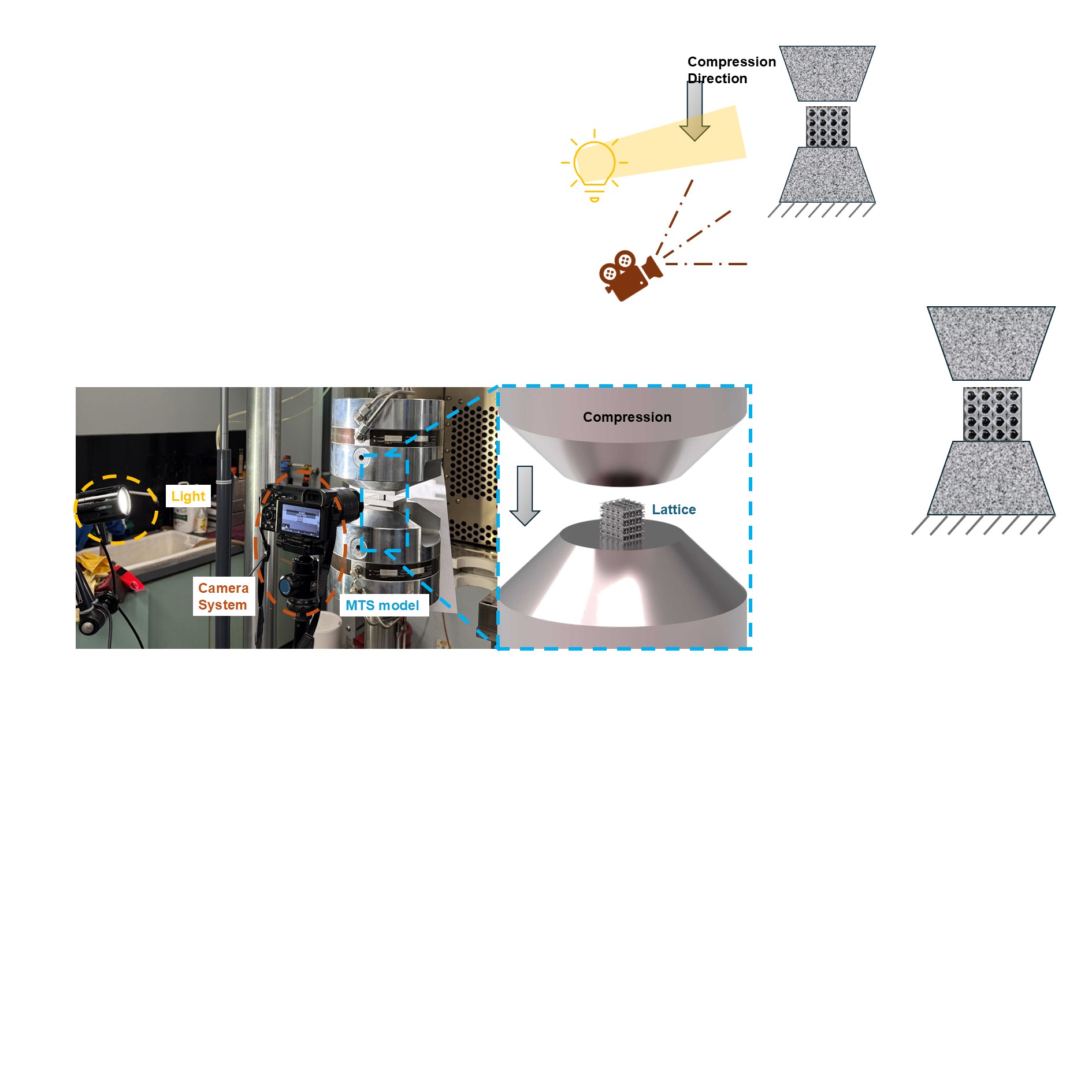


Fig. S4 The schematic of the experimental setup for uniaxial compression test


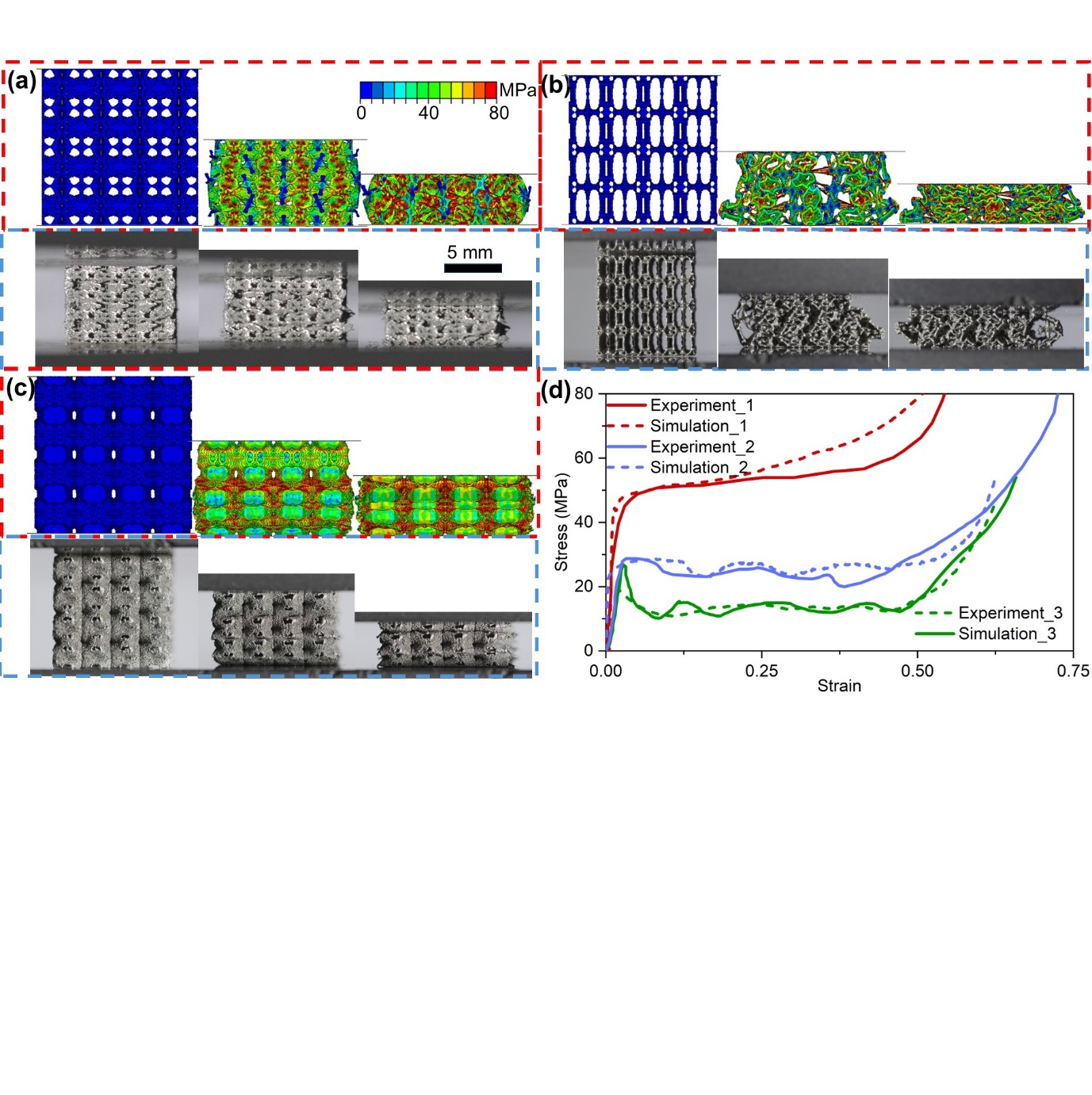


Fig. S5 (a-c) The comparison between FEM models and corresponding experimental tests of uniaxial compression with generated new lattice structures. (d) The compression of stress-strain curves between the numerical models and the experimental results.

Reference

[1] a)T. Okuzono, T. Nitta, K. Sakagami, *Acoustical Science and Technology* **2019**, 40, 221; b)N. Jiménez, J. Groby, V. Romero-García, *Ch. the Transfer Matrix Method in Acoustics, Springer International Publishing, Cham* **2021**, 103.

[2] J. Allard, N. Atalla, *Propagation of sound in porous media: modelling sound absorbing materials 2e*, John Wiley & Sons, **2009**.

[3] X. Li, S. Ding, X. Wang, S. L. A. Tan, W. Zhai, *Advanced Materials Technologies* **2025**, 10, 2400517.

[4] R. K. Abu Al-Rub, D.-W. Lee, K. A. Khan, A. N. Palazotto, *Journal of Engineering Mechanics* **2020**, 146, 04020030.
